# HOMER3 promotes non-small cell lung cancer growth and metastasis primarily through GABPB1-mediated mitochondrial metabolism

**DOI:** 10.1038/s41419-023-06335-5

**Published:** 2023-12-11

**Authors:** Teng Sun, Chao Song, Guoqing Zhao, Shoujie Feng, Jianhao Wei, Lixia Zhang, Xiangming Liu, Zhuoqun Li, Hao Zhang

**Affiliations:** 1 Thoracic Surgery Laboratory, Xuzhou Medical University, Xuzhou, Jiangsu 221006 China; 2 Department of Thoracic Surgery, Affiliated Hospital of Xuzhou Medical University, 99 West Huaihai Road, Xuzhou, 221006 Jiangsu China; 3 Department of Oncology, Shanghai East Hospital, Tongji University School of Medicine, Shanghai, 200120 China

**Keywords:** Cancer metabolism, Non-small-cell lung cancer

## Abstract

Cancer metabolism has emerged as a major target for cancer therapy, while the state of mitochondrial drugs has remained largely unexplored, partly due to an inadequate understanding of various mitochondrial functions in tumor contexts. Here, we report that HOMER3 is highly expressed in non-small cell lung cancer (NSCLC) and is closely correlated with poor prognosis. Lung cancer cells with low levels of HOMER3 are found to show significant mitochondrial dysfunction, thereby suppressing their proliferation and metastasis in vivo and in vitro. At the mechanistic level, we demonstrate that HOMER3 and platelet-activating factor acetylhydrolase 1b catalytic subunit 3 cooperate to upregulate the level of GA-binding protein subunit beta-1 (GABPB1), a key transcription factor involved in mitochondrial biogenesis, to control mitochondrial inner membrane genes and mitochondrial function. Concurrently, low levels of HOMER3 and its downstream target GABPB1 led to mitochondrial dysfunction and decreased proliferation and invasive activity of lung cancer cells, which raises the possibility that targeting mitochondrial synthesis is an important and promising therapeutic approach for NSCLC.

## Introduction

The improved early detection and diagnosis methods and targeted control interventions for lung cancer have led to the decline of its incidence and mortality, according to data collected by the National Center for Health Statistics [1]. However, the 5-year survival of lung cancer, especially non-small cell lung cancer (NSCLC), is currently less than 20%, and NSCLC is still the leading cause of cancer death [1–3]. To advance the progress of lung cancer treatment, it is necessary to further deepen our understanding of the mechanism of the tumorigenesis of lung cancer. The increased understanding of cancer metabolism brings opportunities and challenges for the development of tumor treatments. Natalya et al. [4] classified known cancer-related metabolic changes into six hallmarks, namely the deregulated uptake of glucose and amino acids, the opportunistic modes of nutrient acquisition, increased demand for nitrogen, the regulation of metabolite-related gene expression, the crosstalk with the microenvironment and use of glycolysis/tricarboxylic acid (TCA) cycle intermediates for biosynthesis and energy production. Although actively proliferating tumors are highly dependent on glycolysis as a glucose catabolism pathway, tumor cells have functional mitochondria and retain the ability to conduct oxidative phosphorylation (OXPHOS) [5, 6]. In fact, besides acting as a major source of ATP, mitochondria also participate in the regulations of redox and calcium homeostasis, transcriptional regulation, programmed cell death and producing anabolic intermediates for biosynthesis [7]. Recent studies have shown that the anti-diabetic drug metformin serves as an anticancer agent by inhibiting the mitochondrial electron transport chain (ETC) complex, and taking metformin reduced the cancer incidence and mortality rates among diabetic patients [8–10]. A study by Jiyoung et al. [11] demonstrated that mitochondrial metabolism can be exploited by targeting BTB domain and CNC homology 1 (BACH1) to sensitize breast cancer and potentially other tumor tissues to mitochondrial inhibitors. These findings indicate that mitochondrial metabolism is an attractive target for cancer therapy.

In fact, metformin, rotenone, oligomycin, and other drugs targeting mitochondrial metabolism are limited by inadequate potency or ‘off-target’ pharmacology. However, novel methods of drugging mitochondrial OXPHOS remain largely unexplored. GA-binding protein transcription factor subunit beta-1 (GABPB1, also known as nuclear respiratory factor 2) is an important ETS family transcription factor that binds and regulates mitochondrial genes that are closely related to electron transport [12]. GABPB1 depletion leads to mitochondrial abnormalities and dysfunctions, including reduced mitochondrial mass, ATP production, oxygen consumption, and mitochondrial protein synthesis [13]. Reported studies have demonstrated that GABPB1 may have carcinogenic or anticancer roles in different tumor contexts and has the potential for use as a therapeutic target in glioblastoma and bladder cancer [14–16]. However, its role in NSCLC is still unclear.

Plasma membrane homer scaffold protein 3 (HOMER3) is often highly expressed in more aggressive primary tumors and distant metastases, and has been reported to be an independent predictor of worst prognosis in bladder cancer [17]. In this study, our findings indicate that HOMER3 is highly expressed in NSCLC and closely correlated with poor prognosis. Moreover, our results also show that HOMER3 is positively correlated with GABPB1, as well as several mitochondrial genes. Therefore, we hypothesize that high level of HOMER3 in NSCLC may activate the expressions of downstream mitochondrial genes by GABPB1, thereby promoting mitochondrial anabolism, and creating the conditions for tumor proliferation and metastasis. This study extends our understanding of the involvement of mitochondrial metabolism in the progression of NSCLC, and reveals a new approach to preventing or overcoming NSCLC by targeting HOMER3.

## Material and methods

### Mice

BALB/c nude mice, 6–8 weeks of age, were purchased from Gempharmatech Co., Ltd. (Nanjing, China) and housed in the specific pathogen-free class animal house of Xuzhou Medical University (Xuzhou, China). All animal experiments conformed to the requirements of the Animal Ethics Committee of Xuzhou Medical University. All animal experiments were approved by the Institutional Animal Care and Use Committee of Xuzhou Medical University (202009A258). No preference in mouse sex was given for any of the studies.

### Cell lines

The human NSCLC cell lines H1299 and A549 were purchased from Fu Heng Biotechnology (Shanghai, China). Cells were cultured in Gibco Dulbecco’s modified Eagle’s medium (DMEM) and RPMI-1640 (Thermo Fisher Scientific Inc., Waltham, MA, USA) supplemented with 10% fetal bovine serum and 1% penicillin-streptomycin solution, in an incubator at 37 °C, with a humidified atmosphere containing 5% CO_2_.

### Clinical samples and immunohistochemical staining

All specimens were collected following the guidelines of the U.S. Health Insurance Portability and Accountability Act (HIPAA) of 1996 and supervised by the ethics committee of the hospital. NSCLC patients admitted to department of thoracic surgery of the affiliated hospital of Xuzhou Medical University between September 2012 and April 2015 who did not receive chemotherapy, radiotherapy or immunotherapy were included in this study. The lung tumor tissues and the corresponding adjacent non-tumorous tissues were harvested and fixed in 10% formalin solution after surgery. Ultimately, 129 pathologically confirmed NSCLC tissue samples were used to construct a tissue microarray (TMA). The clinical information of the patients includes age, sex, lymph node metastasis, TNM stage, and prognostic (survival rate).

After heat-mediated antigen retrieval, the microarray was blocked with goat serum for 30 min before adding the primary antibody. The microarray was then incubated with primary anti-HOMER3 antibody for 12 h at 4 °C. Subsequently, the microarray was incubated with horseradish peroxidase (HRP) polymer-conjugated secondary antibody and stained with 3, 3-diaminobenzidine solution and hematoxylin. The staining intensity score was rated as previously reported [18]. Briefly, the immunohistochemical staining intensity scores of HOMER3 were evaluated by combining the percentage of immunostained cells with the staining intensity. The intensity of immunoreacted HOMER3 was scored using a 0–3 scale (0: negative; 1: weak; 2: moderate; 3: strong), and the percentage of immunoreactive cells was graded as 1 (0–25%), 2 (26–50%), 3 (51–75%), and 4 (76–100%). The staining index value (values, 0–12) was determined by multiplying the score for staining intensity by the score for the positive area. For statistical analysis, the level of HOMER3 expression was categorized as low (0–4), middle [4–8] and high [8–12] expression level. Two pathologists independently assessed the staining intensity score under blinded experimental conditions.

### Construction of plasmids, lentivirus, and stable cells

Short hairpin RNAs (shRNAs) and recombinant lentiviruses (HOMER3-LV, PAFAH1B3-LV, GABPB1-LV) were designed and synthesized by GeneChem Co., Ltd. (Shanghai, China). The shRNA-control vector and shRNA*-HOMER3*, and shRNA*-GABPB1* vectors were transfected into H1299 or A549 cells using Lipofectamine 2000 transfection reagent (Invitrogen, Carlsbad, CA, USA). Cells were infected with lentivirus for 24 h and then selected with 2 ng/mL puromycin for 2 weeks, refreshing the medium every 3 days.

### Cell proliferation and apoptosis

The proliferation of H1299 and 549 cells was measured using Celigo cell counting assay, methyl thiazolyl tetrazolium (MTT) cell viability assay and plate clone formation assay. The Annexin V-fluorescein isothiocyanate (FITC) / propidium iodide (PI) apoptosis detection kit (KeyGen Biotech Co., Ltd., Nanjing, China) was used for the detection of apoptotic cells for each treatment.

### Cell invasion, migration, and wound healing assays

For the migration assay, 2 × 10^4^ H1299 or A549 cells were seeded in serum-free medium in the upper chamber (Corning lnc, Corning, NY, USA). For the invasion assay, the upper chamber was coated with Matrigel (Cat No.356237; 1:10 dilution; BD Biosciences, San Jose, CA, USA), and 5 × 10^5^ cells were seeded in serum-free medium in the upper chamber. Cells that traversed the membrane were stained with crystal violet (0.04%) and counted.

### Tumor xenograft study

Twenty mice were randomly assigned to two groups: shControl and sh*HOMER3*. After anesthetizing mice with isoflurane (2.0–3.0%) mixed with air, H1299 cells (5 × 10^6^) were injected subcutaneously into the flanks of mice. Tumor volume (V) was monitored every 3 days by measuring the long axis (L) and the short axis (W) of xenograft tumors and calculated using the following formula: V = (L × W^2^)/2.

### Establishment of lung metastasis model

Luciferase-labeled H1299 cells were injected into BALB/c nude through the tail vein. The fluorescence expression was detected after 1 months, and then the lungs were harvested.

### Co-immunoprecipitation (Co-IP) and mass spectrometry

The co-immunoprecipitation (Co-IP) analysis was performed as previously described [19]. Briefly, protein extracted from H1299 cells was used as input control and for immunoprecipitation. Sample was incubated with anti-Flag antibody and 50 μl agarose A protein overnight at 4 °C. The flag-beads (Millipore-Sigma, Burlington, MA, USA) were washed with lysis buffer and boiled in sodium dodecyl sulfate (SDS) sample buffer, and the pulled-down protein was separated on SDS polyacrylamide gel electrophoresis (PAGE) gels. Then, the gels were stained with Coomassie Blue, and differentially abundant bands were cut out for mass spectrometry (GeneChem Co., Ltd.). Eventually, the supernatants were resolved by Western blotting.

### Liquid chromatography tandem mass spectrometry and parallel reaction monitoring validation

H1299 cells (1.0 × 10^7^) were lysed in SDS-dithiothreitol-tris (SDT) buffer and then boiled for 15 min. After centrifugation at 14,000 × *g* for 40 min, the supernatant was quantified using the bicinchoninic acid (BCA) protein assay kit (Beyotime, Shanghai, China). Then, after acetone precipitation and trypsin digestion, 100 μg peptide mixture of each sample was labeled using the tandem mass tag (TMT) reagent (Thermo Fisher Scientific Inc.) according to the manufacturer’s instructions. The TMT-labeled peptides were fractionated by reversed phase chromatography using the Agilent 1260 infinity II HPLC system (Agilent Technologies Inc., Santa Clara, CA, USA). Liquid chromatography tandem mass spectrometry (LC-MS/MS) analysis was performed on an Orbitrap Exploris 480 Mass Spectrometer (Thermo Fisher Scientific Inc.). Both peptide and protein false discovery rate (FDR) of 1% were enforced using a reverse database search strategy. Proteins with fold change (FC)å 1.2 and *p* value (Student’s *t* test) <0.05) were considered to be differentially expressed proteins. Gene ontology (GO) term and Kyoto Encyclopedia of Genes and Genomes (KEGG) pathway enrichment analyses were performed as previously described [19].

Parallel reaction monitoring (PRM) validation was used to validate the target proteins according to the TMT labeling-based LC-MS/MS analysis, as previously reported [20]. The Skyline (v.3.6) software was used to process and analyze the resulting LC-MS/MS data.

### Western blot analysis

After separation by SDS-PAGE, proteins were transferred to 0.45 μm polyvinylidene fluoride (PVDF) membranes (MilliporeSigma). After blocking with 5% non-fat dry milk for 1 h, primary antibodies against HOMER3, GABPB1, SLC25A22, OPA1, NDUFA3, UQCRC1, ATP5D, COX15 and β-Actin were separately incubated with the membranes at 4 °C overnight. Subsequently, the membranes were incubated with the corresponding secondary antibody for 60 min at room temperature. The antibodies used in this study are listed in Supplementary Table 1.

### Quantitative reverse transcription polymerase chain reaction analysis

Total RNA was extracted using TRIzol reagent and reverse transcription was performed using 2 μg of total RNA. The qRT-PCR analysis was performed according to the procedure described in the UltraSYBR one-step qRT-PCR kit (CWBIO, Beijing, China). The primers used for qRT-PCR analysis are listed in Supplementary Table 2.

### Extracellular flux measurements

The extracellular acidification rate (ECAR) and oxygen consumption rate (OCR) were measured using an Agilent Seahorse XFe96 extracellular flux analyzer (Agilent Technologies Inc.) as previously described [18].

### High-performance liquid chromatography

Cells were quickly extracted with pre-cooled 80% methanol and assayed using a high-performance liquid chromatography (HPLC) system (Agilent Technologies Inc.). ATP levels were calculated by dividing the peak area of samples by standards.

### Quantification and statistical analysis

The sample size in each experiment was chosen to ensure adequate power to detect a pre-specified effect. Studies were not blinded to investigators. The statistical analyses were performed using GraphPad Prism 8.0.1 (GraphPad Software Inc., San Diego, CA, USA). Values are presented as the mean ± SEM unless stated otherwise. Details of the specific statistical analysis are described in the figure legends.

## Results

### HOMER3 is upregulated and associated with a poor prognosis in NSCLC

**Fig. 1:**
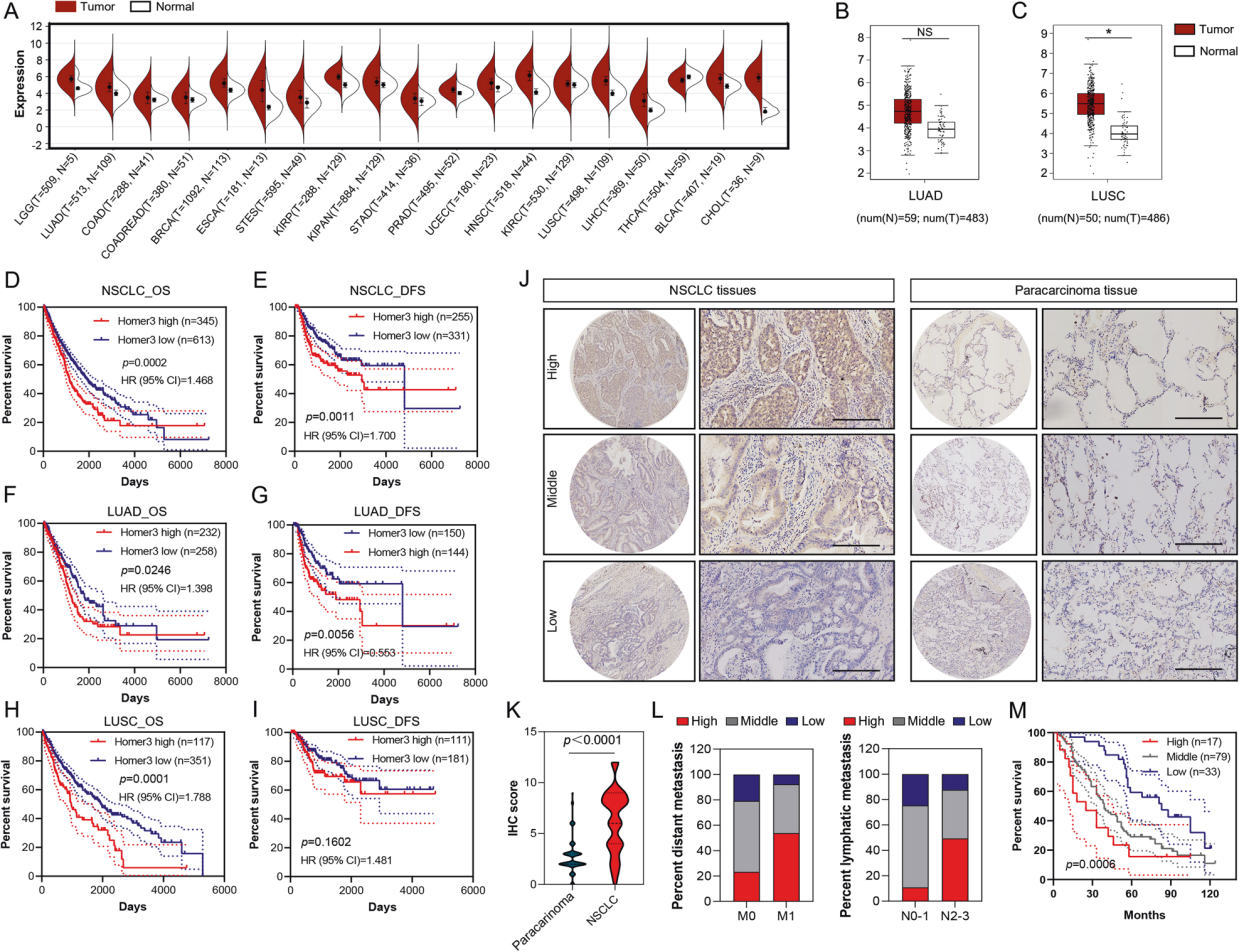
HOMER3 is upregulated and associated with a poor prognosis in NSCLC. **A** The overall HOMER3 expression in multiple human cancers from TCGA database. **B**, **C** HOMER3 expression was analyzed in LUAD (*n* = 483), LUSC (*n* = 486) using GEPIA (http://gepia.cancer-pku.cn/detail.php). **D**–**I** Kaplan–Meier survival curve analyses show that NSLCL, LUAD, and LUSC with high levels of HOMER3 have poor overall survival (OS) and disease-free survival (DFS) according to TCGA database. R software package maxstat (Maximally selected rank statistics with sever p-value approximations version: 0.7-25) was used to determine the optimal truncation value of ENSG0000051128 (HOMER3), log-rank test. **J** Representative images of human NSCLC tumors and paracancerous tissue stained with HOMER3, bar = 200 μm. **K**, **L** HOMER3 is highly expressed in NSCLC and is associated with distant metastasis and lymph metastasis in 129 NSCLC patients, Chi-square test. **M** Kaplan–Meier survival curve analysis was performed to examine the effects of HOMER3 on the survival rate in NSCLC (*n* = 129), log-rank test.

Analysis of The Cancer Genome Atlas (TCGA) datasets revealed that HOMER3 was upregulated in most malignant tumors, including low-grade glioma, breast invasive carcinoma, esophageal carcinoma, head and neck squamous cell carcinoma, cholangio carcinoma (bile duct cancer), liver hepatocellular carcinoma, lung adenocarcinoma (LUAD) and lung squamous cell carcinoma (LUSC) (Fig. 1A–C). Kaplan–Meier survival curve analyses showed that both LUAD and LUSC patients with high levels of HOMER3 had worse survival prognosis (Fig. 1D–I).

Moreover, immunohistochemical analysis on a TMA with 129 human NSCLC specimens further confirmed that HOMER3 was highly expressed in NSCLC at the protein level (Fig. 1J, K). We also found that HOMER3 level was significantly associated with distant metastasis and lymph metastasis (Fig. 1L). Kaplan–Meier survival analysis showed that NSCLC patients with high levels of HOMER3 have poor survival prognosis (Fig. 1M).

### HOMER3 knockdown inhibits NSCLC cell proliferation in vitro and in vivo

**Fig. 2:**
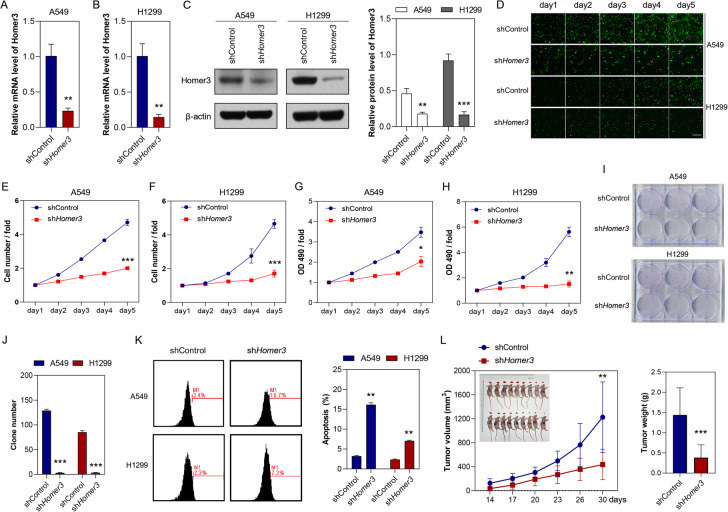
HOMER3 knockdown inhibits NSCLC cell proliferation in vitro and in vivo. **A**–**C** Knockdown of HOMER3 is confirmed at the mRNA and protein level in A549 and H1299 cells. **D** Representative images of Celigo cell counting assay, bar=100 μm. **E**, **F** Quantitative statistics of Celigo cell counting assay in A549 and H1299 cells. **G**, **H** MTT assay of cell proliferation in H1299 and A549 cells. **I**, **J** Colony formation assay for HOMER3 knockdown in A549 and H1299 cells. **K** Flow cytometric analysis of cells apoptosis in A549 and H1299 cells (*n* = 3). **L** The effects of HOMER3 knockdown on tumor volume and weight are assessed using the mouse xenograft model (*n* = 10). *P < 0.05, **P < 0.01, ***P < 0.001 vs. the shControl group, two-tailed Student’s *t*-test.

The basal expressions of HOMER3 in Beas-2b, A549, H1299, H1975, and H226 cell lines was determined by qRT-PCR analysis (Fig. S1A). In addition, shRNA-mediated knockdown of HOMER3 was performed in H1299 and A549 cells (Fig. S1B, Fig. 2A–C). Celigo cell counting assay (Fig. 2B–F), MTT cell viability assay (Fig. 2G, H) and plate clone formation assay (Fig. 2I, J) were performed to determine the role of HOMER3 in the proliferation of A549 and H1299 cells. The results indicated that HOMER3 knockdown significantly inhibited the proliferation of lung cancer cells. Also, the effects of sh*HOMER3* on cell apoptosis were examined by flow cytometry, and the results showed that HOMER3 knockdown promoted the apoptosis of A549 and H1299 cells (Fig. 2K). In addition, we further constructed a mouse xenograft models by subcutaneous injection of treated H1299 cells, and the result showed that sh*HOMER3* treatment led to significantly decreased tumor weight and tumor volume (Fig. 2L).

### HOMER3 knockdown suppresses lung cancer metastasis

**Fig. 3:**
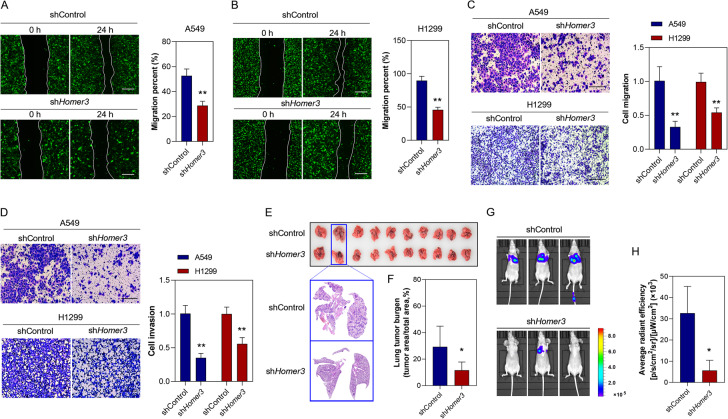
HOMER3 knockdown suppresses lung cancer metastasis. **A**, **B** The wound healing assay shows that HOMER3 knockdown suppresses cell migration in A549 and H1299 cells (*n* = 5), bar=100 μm. **C**, **D** Representative images of cell migration and invasion assays and quantitative data in A549 and H1299 cells with and without sh*HOMER3* treatment (*n* = 5), bar = 50 μm. **E** Representative lung photos and lung sections of tumor-bearing mice. **F** HOMER3 knockdown decreases lung tumor burden in mice four weeks after i.v. injection of H1299 cells (*n* = 10). **G**, **H** The bioluminescence images and quantitative statistical results of mice four weeks after H1299 cells injection (*n* = 5). *P < 0.05, **P < 0.01 vs. the shControl group, two-tailed Student’s *t*-test.

To determine the role of HOMER3 in lung cancer metastasis, we performed scratch assay, cell migration assay and cell invasion assay in A549 and H1299 cells. The results showed that A549 and H1299 cells treated with sh*HOMER3* had reduced invasive and migratory abilities (Fig. 3A–D). In another experiment, luciferase-labeled H1299 cells were injected in to Balb/c nude mice to exam the effect of HOMER3 knockdown on lung cancer metastasis in vivo. Histological examination of the mice showed that low level of HOMER3 prevented H1299 cells from metastasizing to the lung and reduced the primary tumor burden (Fig. 3E, F). The results of a luciferase reporter assay further confirmed the pro-metastatic effect of HOMER3 (Fig. 3G, H).

### HOMER3 can bind PAFAH1B3 and participate in the malignant phenotype of NSCLC cells

**Fig. 4:**
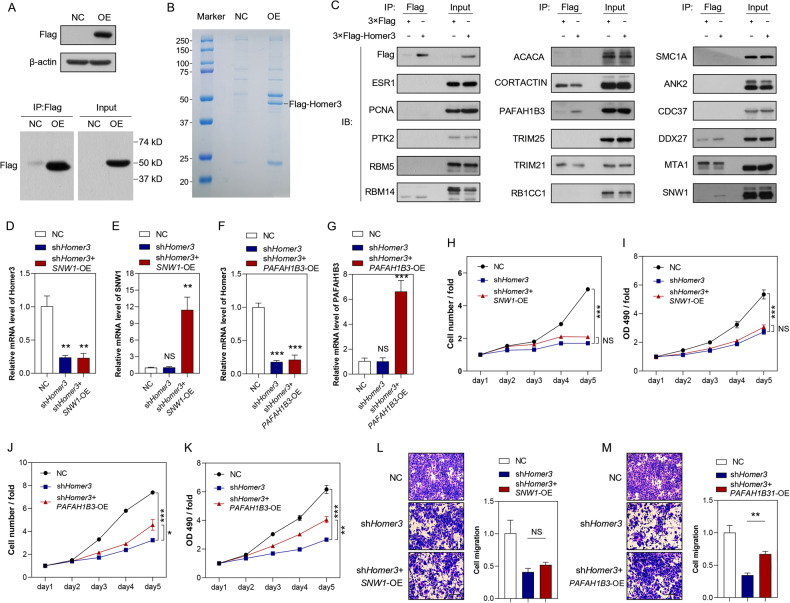
HOMER3 binds to PAFAH1B3 and participates in the malignant phenotype of NSCLC cells. **A** 3 × Flag-HOMER3 was successfully over-expressed in H1299 cells. **B** The image of SDS-PAGE gel stained with Coomassie Blue. **C** Immunoprecipitation analysis was performed to identify the potential targets of HOMER3. **D**, **E** SNW1 was over-expressed in H1299 cells treated with sh*HOMER3*. The levels of HOMER3 and SNW1 were determined by qRT-PCR analysis of H1299 cells. **F**, **G** PAFAH1B3 was overexpressed in H1299 cells treated with sh*HOMER3*. The levels of HOMER3 and PAFAH1B3 were determined by qRT-PCR analysis of H1299 cells. **H**, **I** The effects of *SNW1*-overexpression on the proliferation of H1299 cells with sh*HOMER3* treatment were determined by the Celigo cell counting assay and MTT cell viability assay. **J**, **K** The effects of *PAFAH1B3*-overexpression on the proliferation of H1299 cells with sh*HOMER3* treatment are determined by Celigo cell counting assay and MTT cell viability assay. **L**, **M** The migration and invasion of *SNW1*-overexpressing or *PAFAH1B3*-overexpressing H1299 cells treated with sh*HOMER3* (*n* = 3), bar = 50 μm. **P < 0.01, ***P < 0.001, NS, På 0.05 vs. the Control group, one-way ANOVA followed by the Tukey’s post hoc test.

HOMER3, as a scaffold platform, usually needs to bind with other proteins to perform its biological functions. Therefore, to determine whether HOMER3 regulates the proliferation, apoptosis and invasion of lung cancer cells by binding to other proteins, and to identify HOMER3-binding proteins, we performed Co-IP followed with LC-MS/MS in H1299 cells with overexpressing HOMER3 and control cells (Fig. 4A, B). Compared to control cells, we found 169 proteins with highly affinity binding to HOMER3 in H1299 cells with overexpressing HOMER3 (Supplementary Table 3). Based on the results of the protein interaction network analysis, we selected 17 proteins, that were previously reported to participate in tumor progression, as the potential targets to mediate the biological function of HOMER3 (Fig. S3). The immunoprecipitation results showed that Platelet-activating factor acetylhydrolase IB subunit alpha1 (PAFAH1B3) and SNW domain-containing protein 1 (SNW1) showed increased binding to HOMER3 in H1299 cells with overexpressing HOMER3 (Fig. 4C). To further establish the roles of PAFAH1B3 and SNW1 in lung cancer, PAFAH1B3, and SNW1 were overexpressed in H1299 cells treated with sh*HOMER3* (Fig. 4D–G). The results of the Celigo cell counting assay and MTT cell viability assay showed that *PAFAH1B3*-overexpression, rather than *SNW1*-overexpression, partially restored the proliferation inhibitory effect on H1299 cells induced by HOMER3 knockdown with sh*HOMER3* (Fig. 4H–K). Moreover, *PAFAH1B3*-overexpression has also been shown to partially reverse the reduced invasion and migration abilities of H1299 cells caused by HOMER3 knockdown (Fig. 4L, M).

### Proteomic profiling of *PAFAH1B3*-overexpressing H1299 cells with HOMER3 knockdown

**Fig. 5:**
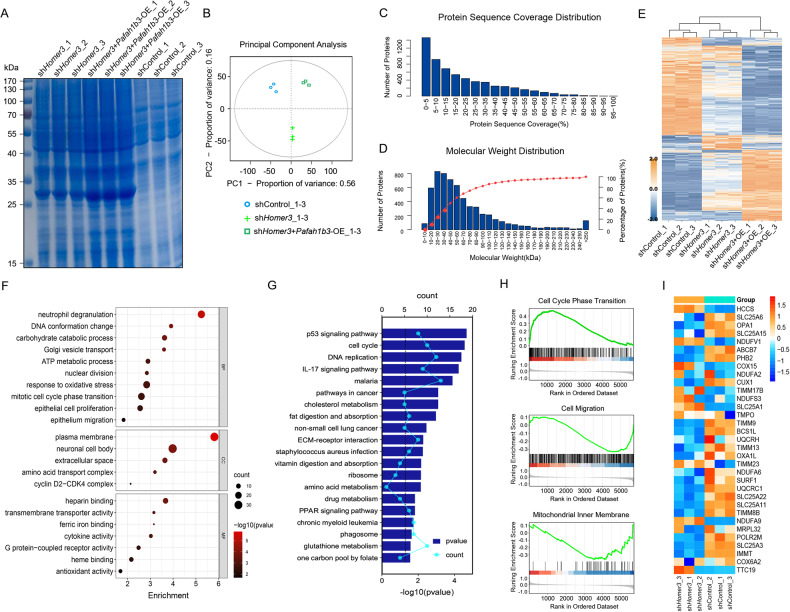
Proteomic profiling of *PAFAH1B3*-overexpressing H1299 cells with HOMER3 knockdown. **A** The image of a SDS-PAGE gel stained with Coomassie Blue. **B** TMT-based quantitative proteomics analysis was performed and the PCA reveals the intra-group and inter-group differences of controls, H1299 cells treated with sh*HOMER3*, and *PAFAH1B3*-overexpressing H1299 cells treated with sh*HOMER3*. **C**, **D** The protein sequence coverage distribution and the relative molecular weight distribution of the samples. **E** Hierarchical clustering showing differentially expressed proteins in H1299 cells with corresponding treatments. **F** GO term enrichment analysis of 45 proteins that are upregulated in the sh*HOMER3*-treated group and downregulated in the sh*HOMER3*-treated + *PAFAH1B3*-overexpression group. **G** KEGG pathway enrichment analysis of 45 proteins that are upregulated in the sh*HOMER3*-treated group and downregulated in the sh*HOMER3*-treated + *PAFAH1B3*-overexpression group. **H** Gene set enrichment analysis identified proteins with normalized enrichment score (NES) and false-discovery rate (FDR) Q value. **I** Heatmap depicting changes in the expression of proteins involved in “mitochondrial inner membrane”.

To elucidate the detailed mechanism through which HOMER3 and PAFAH1B3 regulate the proliferation and invasion of lung cancer, a TMT-based quantitative proteomics analysis was performed on controls, H1299 cells treated with sh*HOMER3*, and *PAFAH1B3*-overexpressing H1299 cells treated with sh*HOMER3*, and identified 6, 048 proteins. The results of the SDS-PAGE analysis and principal component analysis (PCA) indicated that there was an acceptable number of intra-group consistencies and inter-group differences (Fig. 5A, B). The protein sequence coverage distribution and molecular weight distribution are displayed in Fig. 5C, D. An FDR cut-off of 5% and a 1.2-fold change threshold were used to define significantly differentially expressed proteins. Overall, 45 proteins were upregulated in the sh*HOMER3*-treated group and downregulated in the *PAFAH1B3*-overexpressing sh*HOMER3*-treated group. GO term and KEGG pathway enrichment analysis showed that the 45 proteins were enriched in “carbohydrate catabolic process”, “ATP metabolic process”, “epithelial cell proliferation” and “non-small cell lung cancer” (Fig. 5F, G). Gene Set Enrichment Analysis (GSEA) analysis revealed that, compared with control cells, H1299 cells with HOMER3 knockdown had reduced “mitochondrial inner membrane” and “cell migration” related proteins (Fig. 5H). Heatmap showed that proteins-related to mitochondrial inner membrane were markedly downregulated in H1299 cells with HOMER3 knockdown treatment (Fig. 5I).

**Fig. 6:**
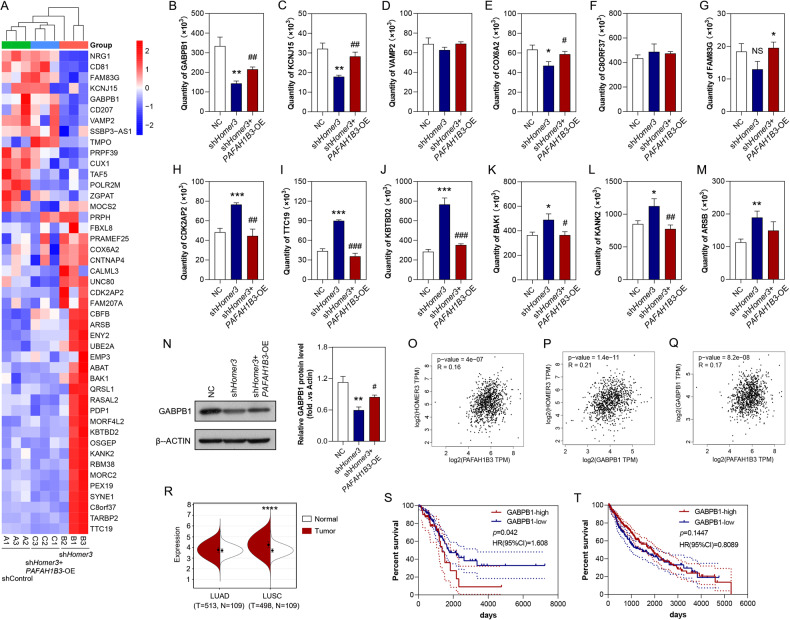
GABPB1 is the potential target of HOMER3 and PAFAH1B3. **A** Heatmap of 45 proteins that are upregulated in the sh*HOMER3*-treated group and downregulated in the sh*HOMER3*-treated + *PAFAH1B3*-overexpression group based on the proteomics data. **A**–**M** Expression of 12 candidate proteins by TMT label-based LC-MS/MS and PRM validation (*n* = 3). **N** Western blot analysis of the expression of GABPB1 in H1299 cell with corresponding treatments (*n* = 3). *P < 0.05, **P < 0.01, ***P < 0.001 vs. the control group; #*P* < 0.05, ##*P* < 0.01, ##*P* < 0.001 vs the sh*HOMER3*-treated group, one-way ANOVA followed by the Tukey’s post hoc test. **O**–**Q** Covariation between mRNA expression of GABPB1, HOMER3, and PAFAH1B3 in NSCLC according to TCGA database. **R** GABPB1 is highly expressed in LUSC according to TCGA database. **S** Kaplan–Meier survival curve analysis showed that GABPB1 is closely related to the poor prognosis of LUAD, log-rank test. **T** Kaplan–Meier survival curve analysis showed that there is no significant correlation between the expression level of GABPB1 and the prognosis of LUSC patients, log-rank test.

We then selected 12 proteins from these 45 identified differentially expressed proteins for parallel reaction monitoring. The results indicated that the levels of GABPB1, KCNJ15, COX6A2, CDK2AP2, TTC19, KBTBD2, BAK1, and KANK2 were regulated by HOMER3 knockdown and *PAFAH1B3*-overexpression (Fig. 6A–M). Notably, *GABPB1*, a key gene involved in mitochondrial biogenesis and function, was further confirmed to be regulated by HOMER3 and PAFAH1B3 (Fig. 6N). Moreover, a positive correlation between GABPB1 and HOMER3, and between GABPB1 and PAFAH1B3 were observed in NSCLC (Fig. 6O–Q). TCGA datasets also showed that GABPB1 was upregulated in LUSC and was associated with poor prognosis of in LUAD.

### GABPB1 deficiency leads to mitochondrial dysfunction

**Fig. 7:**
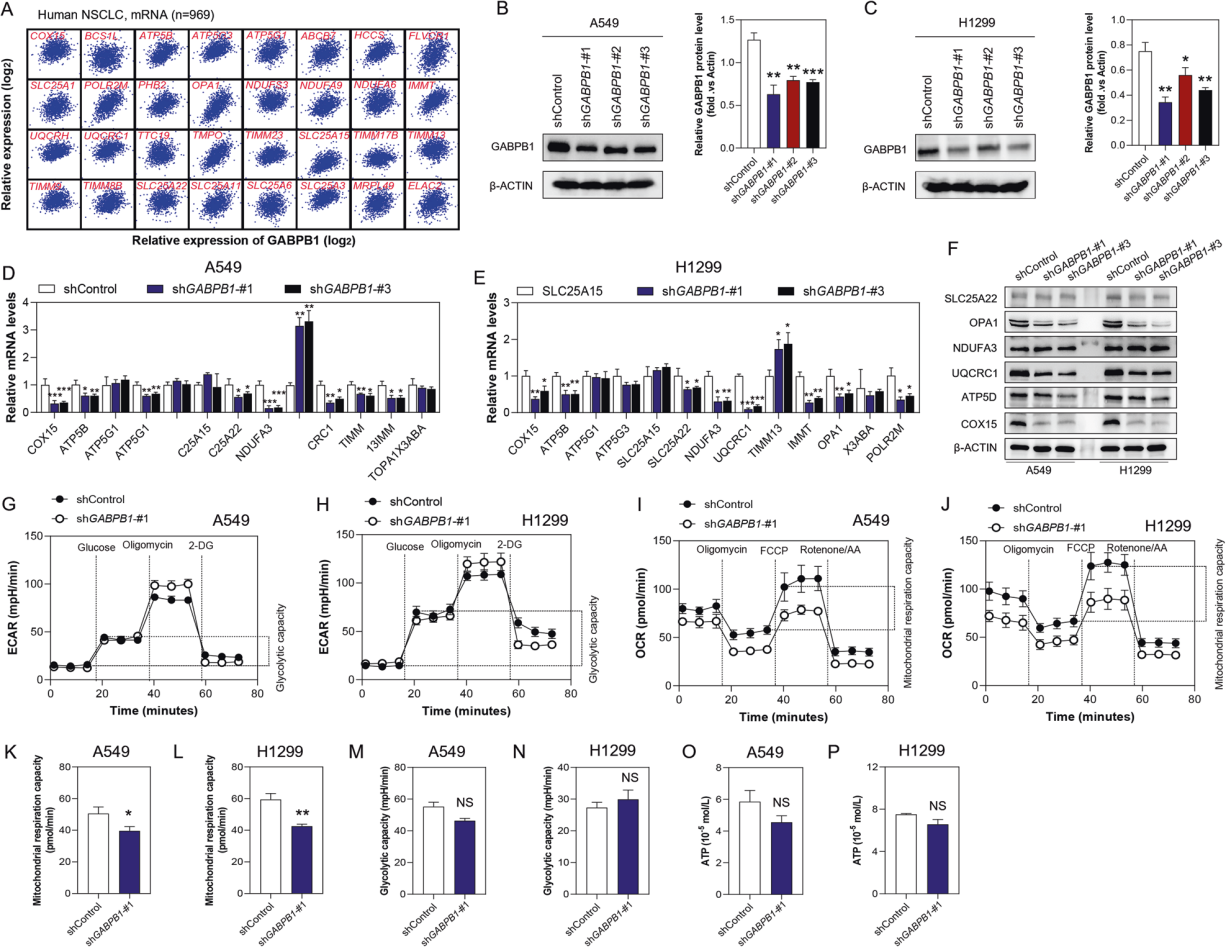
GABPB1 deficiency leads to mitochondrial dysfunction. **A** Covariation between mRNA expression of GABPB1 (*x* axis) and indicated mitochondrial genes (*y* axis) in NSCLC in TCGA database. **B**, **C** The gene transfection efficiency was determined by Western blot analysis in A549 and H1299 cells. **D**, **E** The expression levels of genes related to “mitochondrial inner membrane” are determined by qRT-PCR analysis in A549 and H1299 cells. **F** Western blot analysis revealed that the levels of OPA1, UQCRC1, ATP5D, COX15 are reduced in H1299 cells treated with sh*GABPB1*. **G**–**J** Measurements of the ECAR and OCR in A549 and H1299 cells treated with sh*GABPB1* treatment. **K**–**N** Effects of GABPB1 knockdown on the mitochondrial respiration capacity and glycolytic capacity in A549 and H1299 cells. **O**, **P** High-performance liquid chromatography (HPLC) analysis for ATP in A549 and H1299 cells (*n* = 3). *P < 0.05, **P < 0.01, ***P < 0.001, NS, På 0.05, vs. the shControl group, one-way ANOVA followed by the Tukey’s post hoc test or two-tailed Student’s *t*-test.

Analysis of TCGA datasets also indicated a significant positive correlation between the expression of *GABPB1* and genes related to “mitochondrial inner membrane”, including *COX15*, *ATP5B*, *ATP5G3*, *HCCS*, *FLVCR1*, *OPA1*, X3ABA et al. In addition, we also successfully knocked down the expression of GABPB1 in H1299 cells (Fig. 7B, C). The results of the qRT-qPCR and western blot analysis indicated that GABPB1 knockdown decreased the levels of COX15, ATP5D, ATP5G3, NDUFA3, UQCRC1, OPA1 and SLC25A22 in A549 and H1299 cells (Fig. 7D–F). We also determined whether the GABPB1-induced changes in mitochondrial inner membrane genes affect the mitochondrial function of lung cancer cells by measuring both the OCR and ECAR. A549 and H1299 cells with GABPB1 knockdown exhibited decreased basal and maximum OCR and mitochondrial respiration capacity, moderately increased maximum ECAR, and unchanged glycolytic capacity (Fig. 7G–N). However, HPLC analysis revealed that knockdown of GABPB1 did not significantly reduce ATP production in A549 and H1299 cells (Fig. 7O, P). These results diminish the effect of the role of ATP production in the regulations of HOMER3 or GABPB1 on the proliferation and invasion of lung cancer cells.

Besides supplying ATP, the TCA cycle also provides metabolic precursors for the biosynthesis of nonessential amino acids including aspartate (Asp). Our results showed that additional supplementation with malate (Mal), oxaloacetic acid (Oaa) or Asp instead of α-ketoglutarate (α-KG) can significantly reverse the proliferation inhibitory effect of knockdown of GABPB1 on H1299 cells. These findings suggest that mitochondrial-dependent Asp synthesis may mediate the regulation of lung cancer cell proliferation by GABPB1.

### GABPB1 contributes to the carcinogenic phenotype of cells overexpressing HOMER3 in vitro and in vivo

**Fig. 8:**
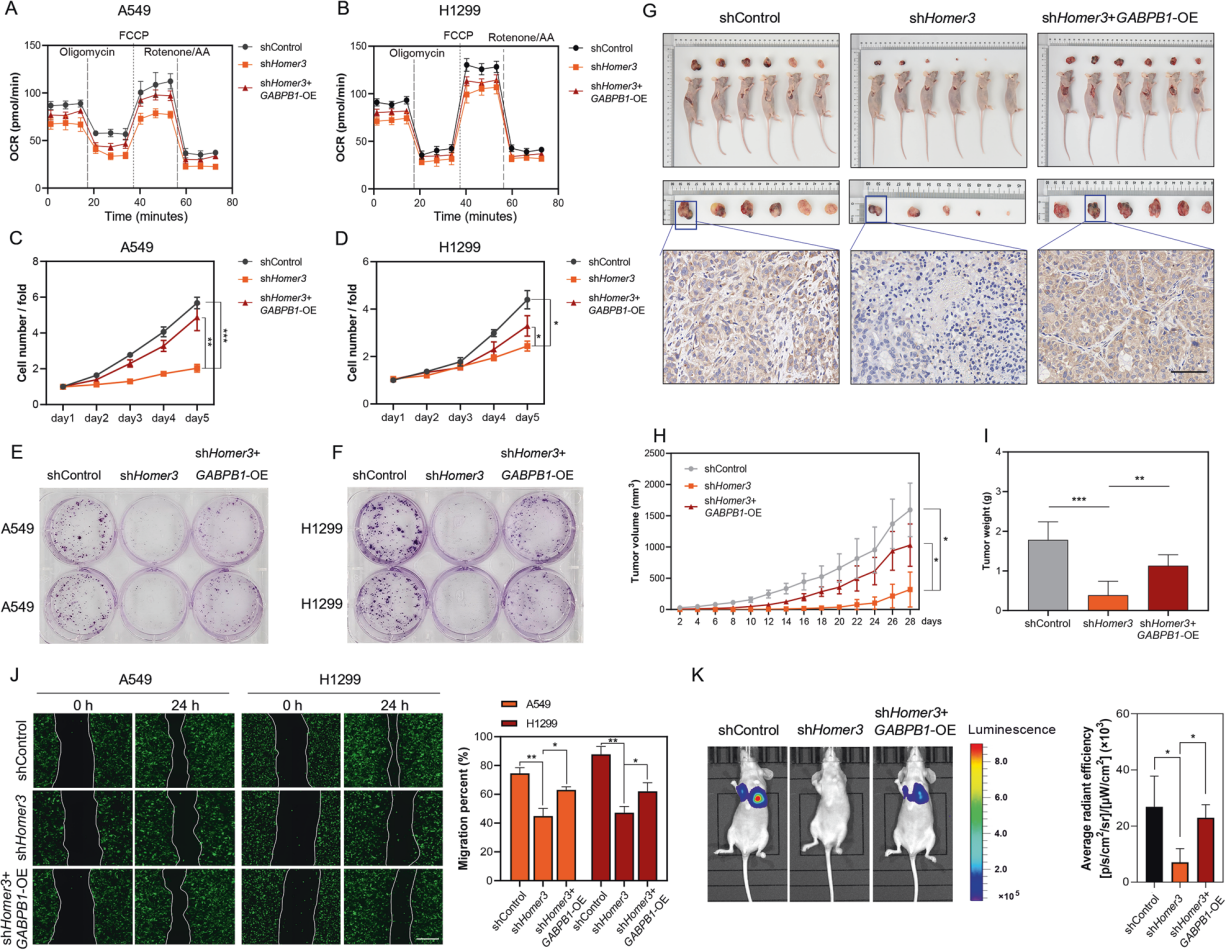
GABPB1 contributes to the carcinogenic phenotype of cells overexpressing HOMER3 in vitro and in vivo. **A**, **B** Measurements of the OCR in A549 and H1299 cells with corresponding treatments. **C**, **D** The effects of *GABPB1*-overexpression on the proliferation of A549 and H1299 cells treated with sh*HOMER3* were determined by the Celigo cell counting assay. **E**, **F** Colony formation assays were performed to determine the effects of *GABPB1*-overexpression on the proliferation of A549 and H1299 cells treated with sh*HOMER3*. **G**–**I** The effects of *GABPB1*-overexpression on tumor volume and weight were assessed using the mouse xenograft model (*n* = 6). **J** The wound healing assay shows that *GABPB1*-overexpression restores the reduced invasiveness caused by HOMER3 knockdown (*n* = 3), bar = 100 μm. **K** The bioluminescence images and quantitative statistical results of mice four weeks after H1299 cells injection (*n* = 5). *P < 0.05, **P < 0.01, ***P < 0.001 vs. the indicated group, one-way ANOVA followed by the Tukey’s post hoc test or two-tailed Student’s *t*-test.

This study also found that knockdown of HOMER3 could reduce the OCR of H1299 and A549 cells, and *GABPB1*-overexpression effectively reversed this process (Fig. 8A, B). As expected, *GABPB1*-overexpression markedly blocked the inhibition of proliferation of A549 and H1299 cells induced by knockdown of HOMER3 in vitro (Fig. 8C–F). The results from the experiments using the mouse xenograft model, developed by subcutaneous injection of treated H1299 cells, showed that knockdown of HOMER3 decreased the expression of GABPB1 in xenografts, and *GABPB1*-overexpression reversed the inhibitory effect of sh*HOMER3* treatment on tumor growth (Fig. 8G–I). Moreover, overexpression of GABPB1 also partially reversed the decrease of invasive ability and lung metastasis of lung cancer cells induced by HOMER3 knockdown (Fig. 8J, K). In general, these findings GABPB1 supports the carcinogenic phenotype of cells overexpressing HOMER3 in vitro and in vivo.

## Discussion

This study revealed that the high expression of HOMER3 greatly contributes to the high proliferative activity and high risk of metastasis of NSCLC, and is closely correlated with poor prognosis. The finding that low levels of HOMER3 and its downstream target GABPB1 led to mitochondrial dysfunction and decreased proliferation and invasive ability of lung cancer cells raises the possibility that targeting mitochondrial synthesis might be an important and promising therapeutic strategy for NSCLC.

HOMER3 is widely expressed in developing organs, including brain, cochlea, liver, and lungs [21]. Previous studies have established the role of HOMER3 in neuropsychological disorders [22]. In the mammalian brain, HOMER3 can bind to several postsynaptic density proteins or postsynaptic scaffolding proteins to regulate the Ca2+ signaling and calcium homeostasis, which are closely related to memory formation, neuronal plasticity, depression, schizophrenia, and addiction to drugs and alcohol [23, 24]. Loukia et al. [25] also showed that the binding and interaction of HOMER3 and amyloid precursor protein played a critical role in Alzheimer’s disease pathology. Remarkably, recent studies reveal that HOMER3 is a potential tumor therapy target. A study by Liu et al. [26] indicated that HOMER3 was selectively overexpressed in triple-negative breast cancer, and served as a scaffold protein to simultaneously interact with β-catenin and promoted the aggressiveness of breast cancer cells. Andreia et al. [17] also found that HOMER3 allowed the identification of the subset of bladder cancer patients with the worst prognosis and hold the potential to address the high aggressiveness of hypoxic bladder cancer cells. The results of this study indicated that HOMER3 is upregulated and associated with a poor prognosis in NSCLC. Knockdown of HOMER3, significantly decreased the proliferation and invasion abilities of lung cancer cells in vitro *and* in vivo. These findings suggest that HOMER3 also serves as a potential therapeutic target to prevent or overcome NSCLC.

An emerging theme is that cancer cells display metabolic plasticity. Indeed, when a tumor cell proliferates vigorously, glucose is preferentially used in the glycolysis pathway, due to a regulated metabolic state to support increased biosynthetic demand, rather than an adaptation to a defect in respiration [27]. However, despite their high glycolytic rates, mitochondrial metabolism is necessary for cancer cell proliferation and tumorigenesis [28, 29]. A recent systematic review and meta-analysis of more than 24,000 participants provided high-quality evidence that metformin could be a useful adjuvant agent, with the greatest benefits seen in patients with colorectal and prostate cancer, particularly in those receiving radical radiotherapy [30]. Kang et al. [31] further confirmed that metformin had independent protective associations with lung cancer mortality, had inverse association with lung cancer risk. A recent preclinical study also showed that targeting mitochondrial genome reduces the respiratory function and tumorigenic potential of breast cancer cells [5]. Clearly, targeting mitochondrial metabolism has emerged as an attractive target for cancer therapy. Our study revealed that the knockdown of HOMER3 led to marked mitochondrial dysfunction in lung cancer cells. Mechanistically, the binding of HOMER3 and PAFAH1B3 activates the transcriptional regulation of mitochondrial genes by GABPB1 in lung cancer cells. In fact, GABPB1 is a master regulator of nuclear-encoded mitochondrial genes, which plays crucial roles in the progression of cell cycle, protein synthesis as well as in mitochondrial biogenesis. HOMER3 knockdown led to decreased expression of GABPB1, as well as many mitochondrial inner membrane genes, which greatly affect the mitochondrial function.

Notably, previous studies have reported that GABPB1 loss modestly reduced the mitochondrial mass, oxygen consumption and ATP production in eukaryotic cells [13, 32]. However, we found that while GABPB1 deletion reduced the OCR of lung cancer cells, it did not significantly affect ATP production. We hypothesize that this may be due to the high glycolysis rate or the compensation of multiple metabolic pathways. Furthermore, additional supplementation with Asp was found to reverse the GABPB1 knockdown-induced decreased proliferation of lung cancer. Asp biosynthesis has recently been shown to be critically dependent on OXPHOS. Lucas et al. [13] proposed that a major role of respiration in proliferating cells is to provide electron acceptors for Asp synthesis, and supplying Asp enables the proliferation of respiration-deficient cells in the absence of exogenous electron acceptors. Therefore, we believe that HOMER3 controls the tumorigenic potential of lung cancer by regulating the GABPB1-dependent mitochondrial Asp synthesis.

In conclusion, this study demonstrated the remarkable efficacy of HOMER3 in regulating the metabolism, proliferation, and metastasis of lung cancer cells. At the mechanistic level, HOMER3 and PAFAH1B3 cooperate to upregulate the level of GABPB1 and control mitochondrial inner membrane genes and mitochondrial function. Low levels of HOMER3 or GABPB1 cause severe mitochondrial dysfunction, thereby inhibiting the growth and metastasis of lung cancer. This study provides a promising strategy for targeting mitochondrial metabolism for the treatment of lung cancer.

### Supplementary information

Original Data File

Supplementary tables and figures

aj-checklist

## Data Availability

The datasets used and/or analyzed in the current study are available from the corresponding author upon reasonable request.
