# Supplementary material for: HOMER3 promotes non-small cell lung cancer growth and metastasis primarily through GABPB1-mediated mitochondrial metabolism
Source: Cell Death Dis. 2023 Dec 11;14(12):814. doi: 10.1038/s41419-023-06335-5 (PMC10713516; doi:10.1038/s41419-023-06335-5)
Supplement: Supplementary file 1 — Original Data File [file 41419_2023_6335_MOESM1_ESM.pptx]

## Slide 1
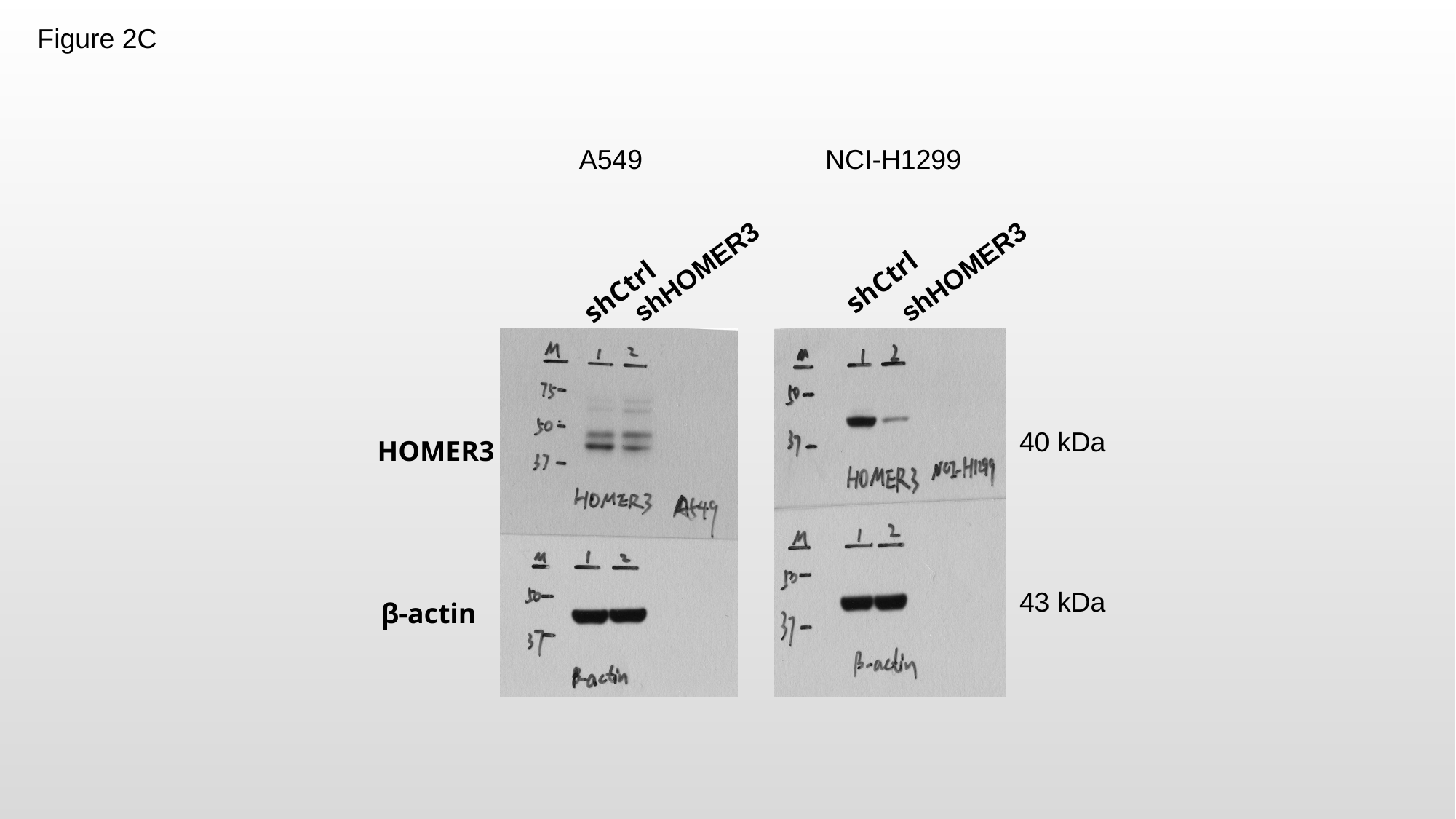

Figure 2C
A549 NCI-H1299
shHOMER3
shHOMER3
shCtrl
shCtrl
HOMER3
β-actin
40 kDa
43 kDa

## Slide 2
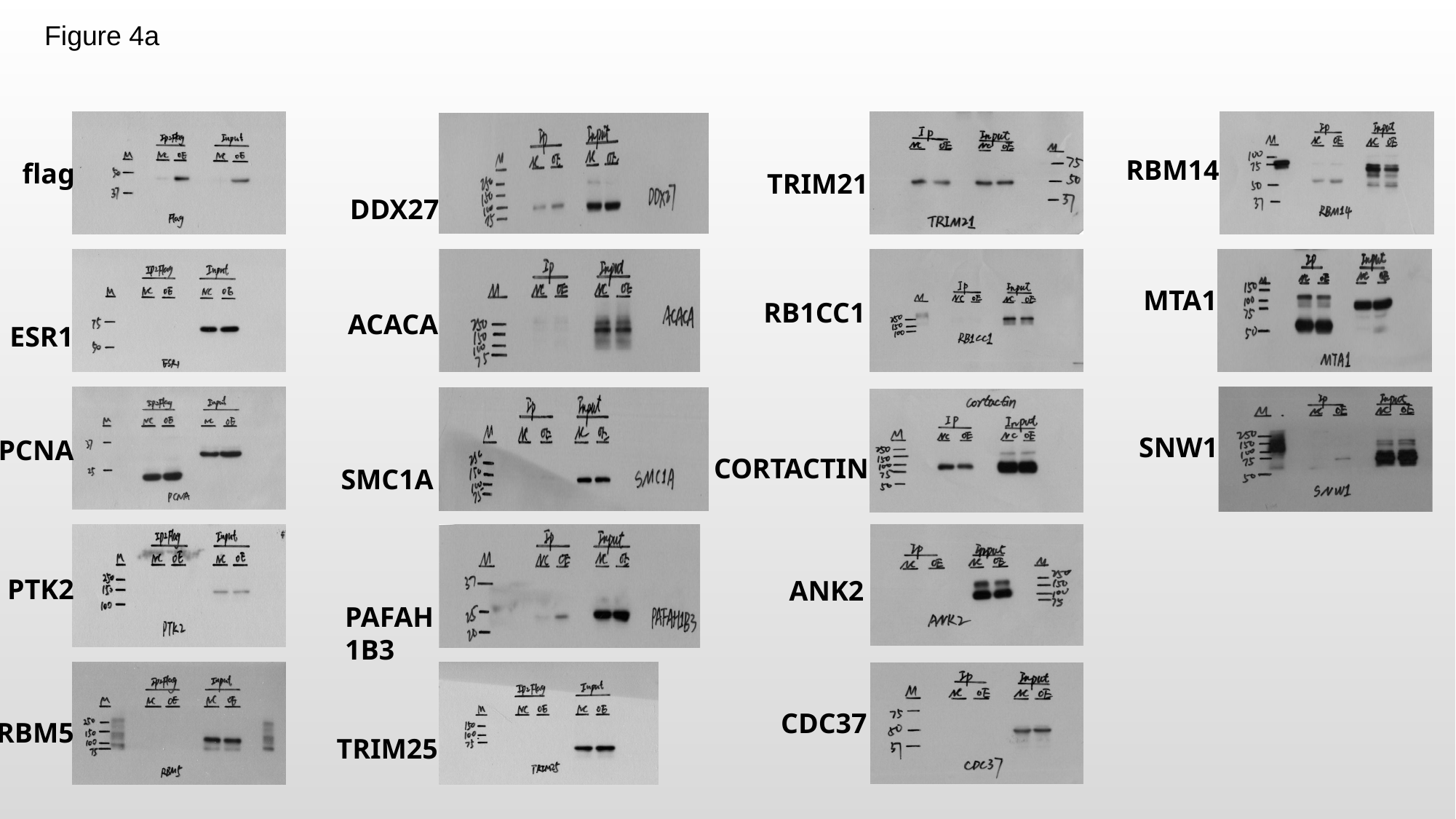

Figure 4a
RBM14
flag
TRIM21
DDX27
MTA1
RB1CC1
ACACA
ESR1
SNW1
PCNA
CORTACTIN
SMC1A
PTK2
ANK2
PAFAH
1B3
CDC37
RBM5
TRIM25

## Slide 3
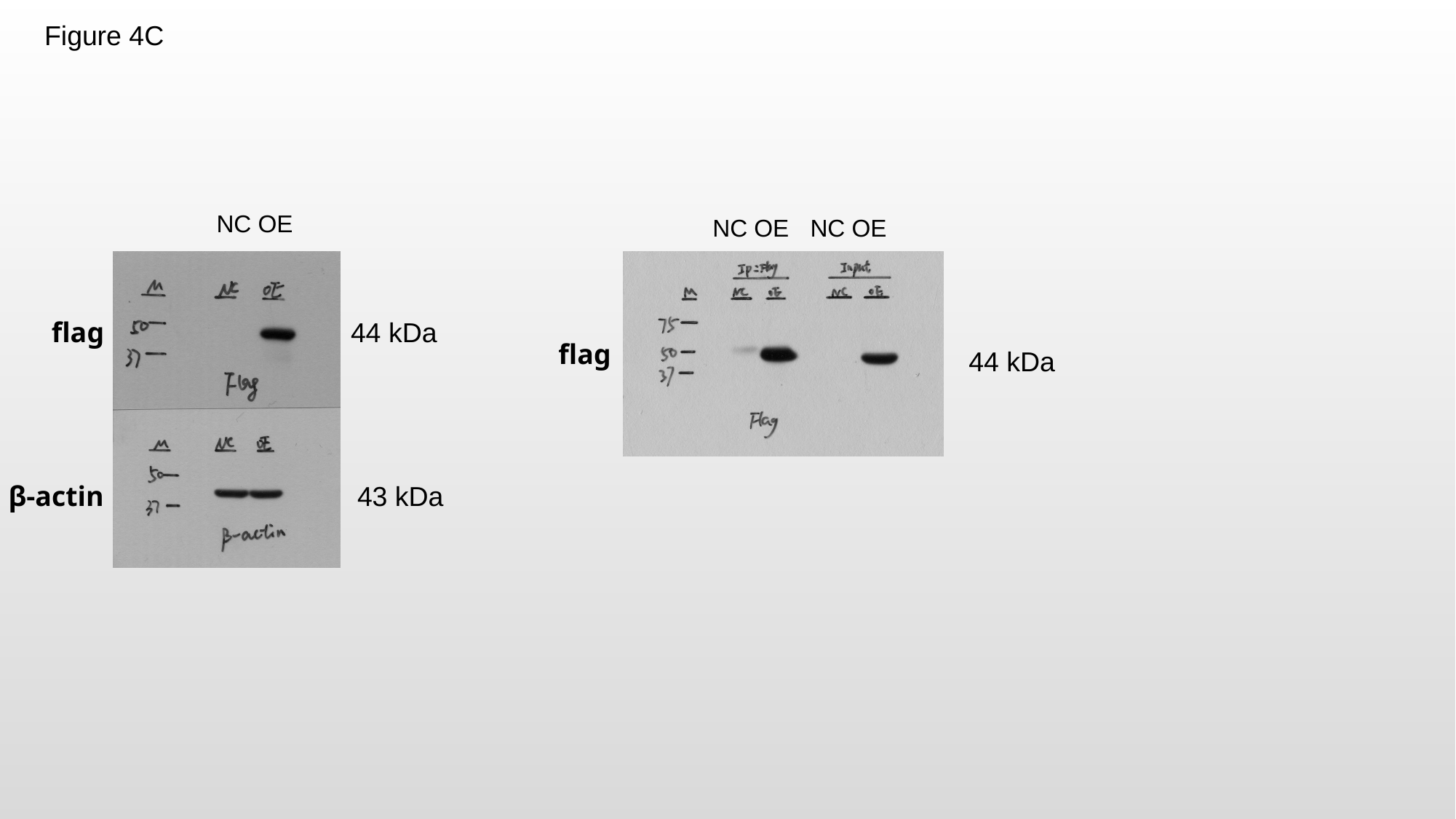

Figure 4C
NC OE
NC OE
NC OE
44 kDa
flag
flag
44 kDa
43 kDa
β-actin

## Slide 4
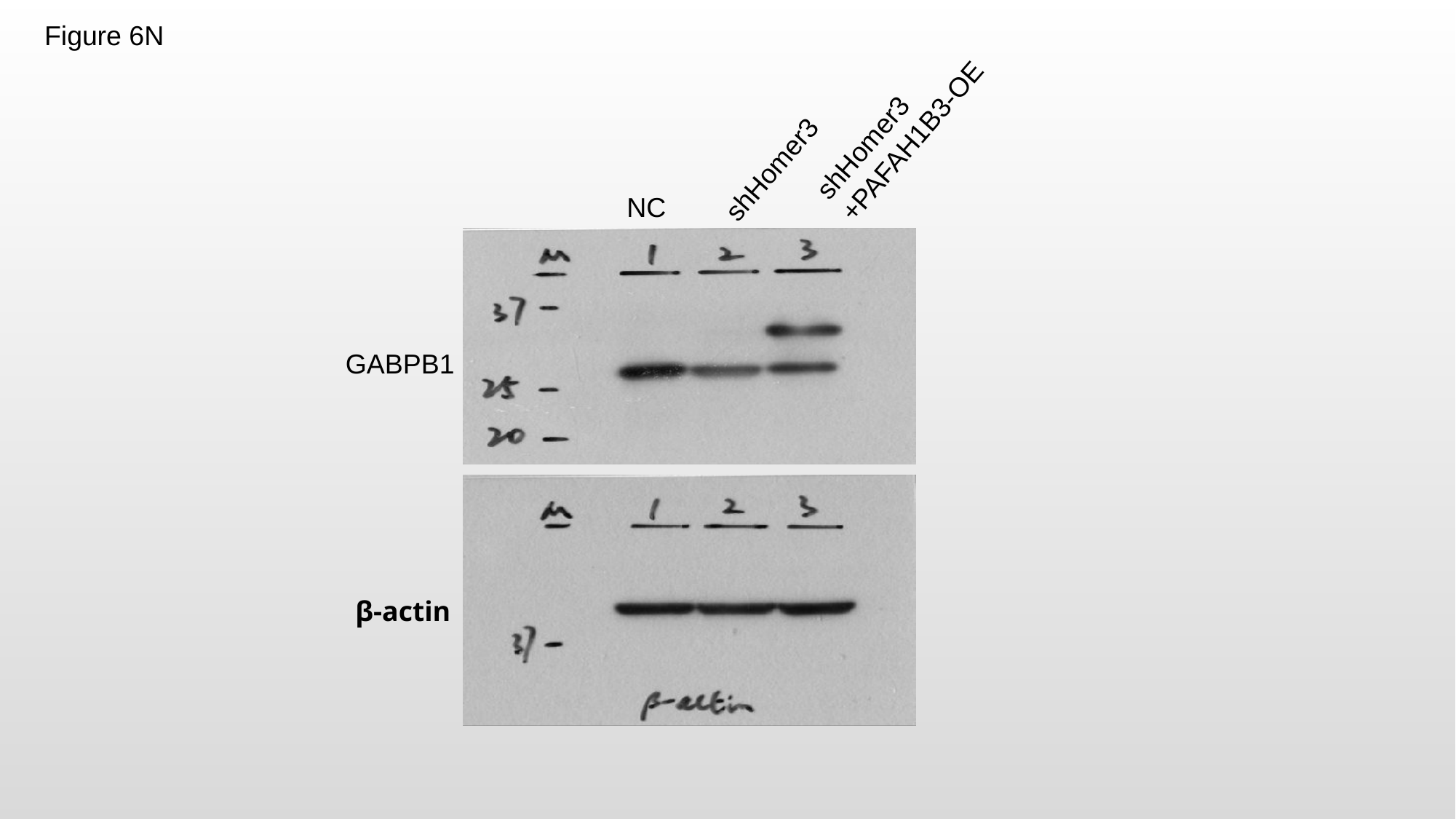

Figure 6N
shHomer3
+PAFAH1B3-OE
shHomer3
NC
GABPB1
β-actin
