# Supplementary material for: HOMER3 promotes non-small cell lung cancer growth and metastasis primarily through GABPB1-mediated mitochondrial metabolism
Source: Cell Death Dis. 2023 Dec 11;14(12):814. doi: 10.1038/s41419-023-06335-5 (PMC10713516; doi:10.1038/s41419-023-06335-5)
Supplement: Supplementary file 2 — Supplementary tables and figures [file 41419_2023_6335_MOESM2_ESM.docx]

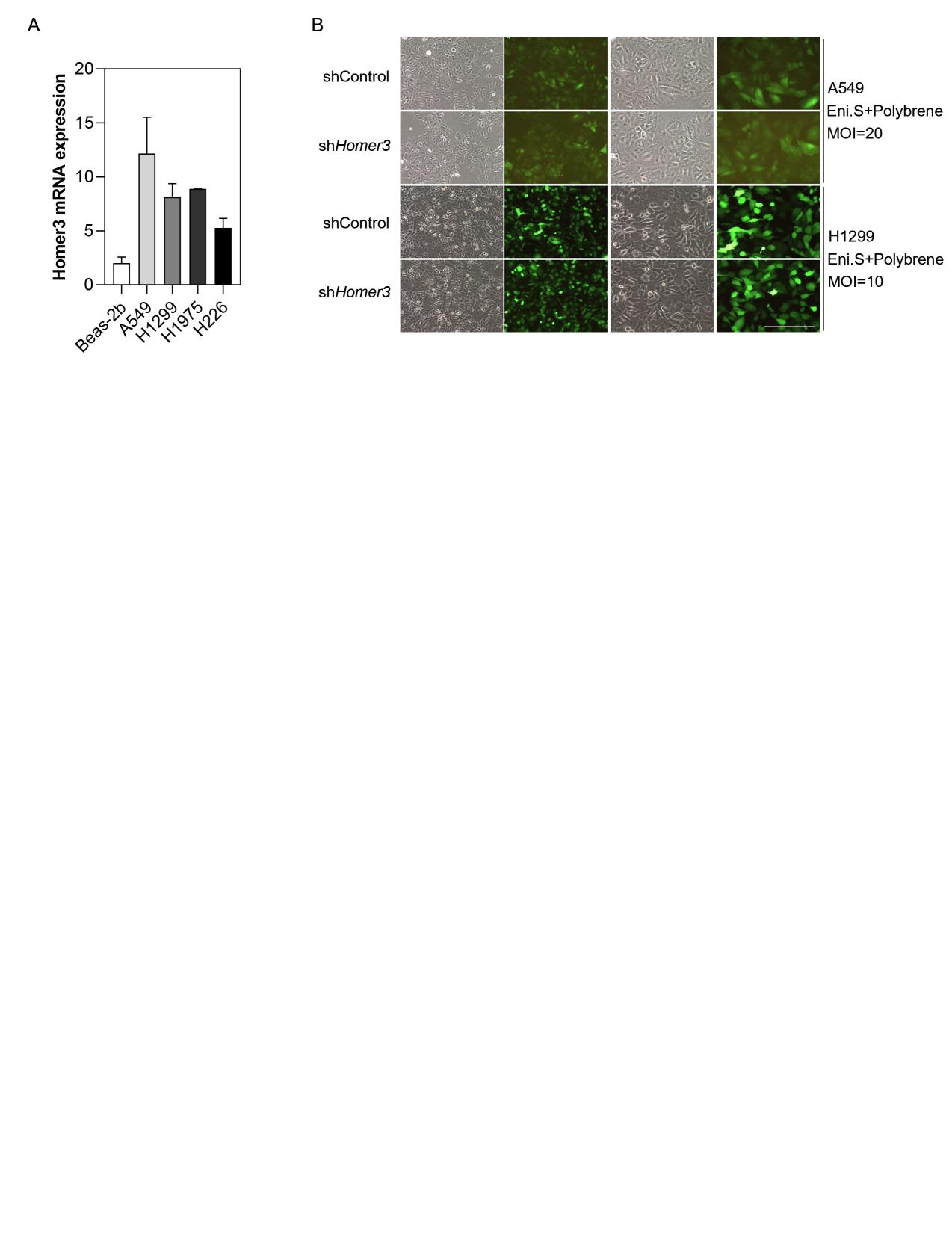
**Figure S1. Determination of the basal expressions of HOMER3.**

1. The basal expressions of HOMER3 in Beas-2b, A549, H1299, H1975 and H226 cell lines are determined by qRT-PCR analysis (n=3).

(B) Representative images of A549 and H1299 cells infected with lentiviruses, bar=50 μm.


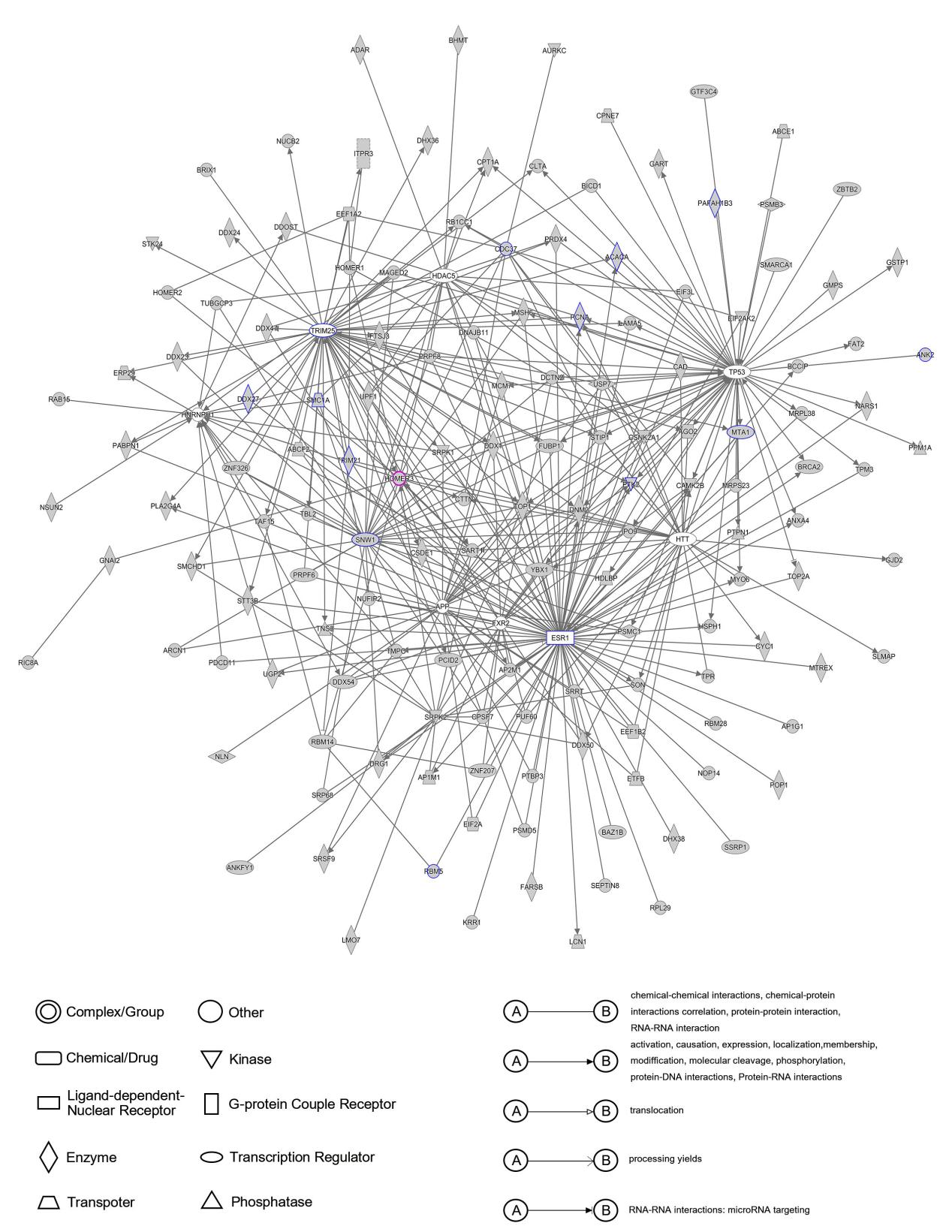


**Figure S2. The gene interaction network diagram.**


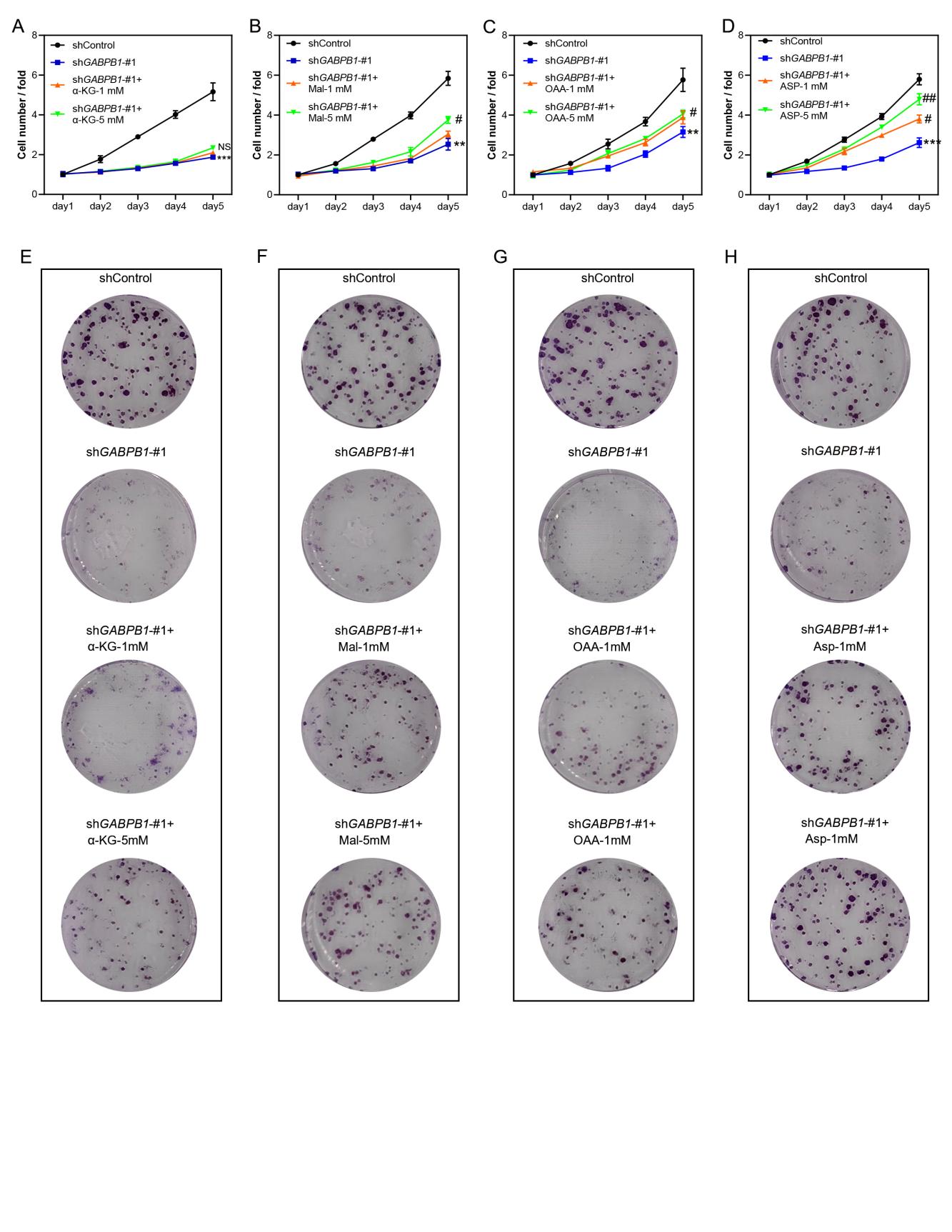


**Figure S3. Additional supplements of ASP, Mal and OAA restore the inhibition of proliferation caused by HOMER3 knockdown.**

1. D) Quantitative statistics of Celigo cell counting assay in H1299 (n=3),**P＜0.01, ***P＜0.001 vs. the shControl group; #P＜0.05, ##P＜0.01, NS, P＞0.05 vs. the shGABPB1-#1 group. One-way ANOVA followed by the Tukey’s post hoc test or two-tailed Student’s t-test.

(E-H) Colony formation assays for H1299 cells with corresponding treatments (n=3).

Supplementary Table 1: Anti-body and reagents used in this study.

| REAGENT or RESOURCE | SOURCE | IDENTIFIER |
| --- | --- | --- |
| Antibodies |  |  |
| Rabbit Polyclonal anti-HOMER3 | Abcam | Cat#ab97438 |
| Rabbit Polyclonal anti-RBM5 | Proteintech | Cat#19930-1-AP |
| Rabbit Polyclonal anti-SNW1 | Proteintech | Cat#25926-1-AP |
| Rabbit Polyclonal anti-PTK2 | Abcam | Cat#ab76496 |
| Rabbit Polyclonal anti-ESR1 | Abcam | Cat#ab108398 |
| Mouse Polyclonal anti-PCNA | CST | Cat#2586 |
| Rabbit Polyclonal anti-TRIM25 | Abcam | Cat#ab167154 |
| Mouse Polyclonal anti-ANK2 | Santa Cruz | Cat#sc-70358 |
| Mouse Monoclonal anti-Flag | Sigma | Cat#F1804 |
| Rabbit Polyclonal anti-TRIM21 | Proteintech | Cat#12108-1-AP |
| Rabbit Monoclonal anti-RB1CC1 | Proteintech | Cat#17250-1-AP |
| Rabbit Monoclonal anti-RBM14 | Proteintech | Cat#10196-1-AP |
| Rabbit Monoclonal anti-CORTACTIN | Proteintech | Cat#10218-1-AP |
| Rabbit Polyclonal anti-CDC37 | Proteintech | Cat#10218-1-AP |
| Mouse Polyclonal anti-MTA1 | Santa Cruz | Cat#sc-373765 |
| Rabbit Polyclonal anti-β-Actin | Proteintech | Cat#20536-1-AP |
| Rabbit Monoclonal anti-PAFAH1B3 | Abcam | Cat#ab170877 |
| Rabbit Polyclonal anti-ACACA | CST | Cat#4190 |
| Rabbit Polyclonal anti-SMC1A | Abcam | Cat#ab133643 |
| Rabbit Monoclonal anti-DDX27 | Sigma | Cat#HPA047087 |
| Rabbit Polyclonal anti-GABPB1 | Proteintech | Cat#12597-1-AP |
| Rabbit Polyclonal anti-SLC25A22 | Proteintech | Cat#25402-1-AP |
| Rabbit Polyclonal anti-OPA1 | Proteintech | Cat#27733-1-AP |
| Rabbit Polyclonal anti-NDUFA3 | Proteintech | Cat#17257-1-AP |
| Rabbit Polyclonal anti-UQCRC1 | Proteintech | Cat#21705-1-AP |
| Rabbit Polyclonal anti-ATP5D | Proteintech | Cat#14893-1-AP |
| Rabbit Polyclonal anti-COX15 | Proteintech | Cat#11441-1-AP |
| Anti-Rabbit IgG | CST | Cat#7074 |
| Anti-Mouse IgG | CST | Cat#7076 |

| Chemicals, Peptides, and Recombinant Protein | |  |
| --- | --- | --- |
| Seahorse XF 1.0 M glucose solution | Aligent | Cat#103577-100 |
| Seahorse XF 100 mM pyruvate solution | Aligent | Cat#103578-100 |
| Seahorse XF 200 mM glutamine solution | Aligent | Cat#103579-100 |
| CCK-8 | KeyGen Biotech | Cat#KGA317 |
| Annexin V-FITC Apoptosis Detection Kit | KeyGEN BioTECH | Cat#KGA108 |
| Seahorse XF Cell Mito Stress Test Kit | Aligent | Cat#103015-100 |
| Seahorse XF Glycolysis Stress Test Kit | Aligent | Cat#103020-100 |
| UltraSYBR One Step RT-qPCR Kit | CWBIO | Cat#CW0659 |
| HiScript Q RT SuperMix for qPCR | Vazyme | Cat#R123-01 |

Supplementary Table 2: Primer sequences used in this study.

| Gene | Forward (5’-3’) | Reverse (5’-3’) |
| --- | --- | --- |
| HOMER3 | AGGGAGCAGCCAATCTTCAG | CCCACTGCCCGAACTTCTG |
| GABPB1 | TCCACTTCATCTAGCAGCACA | GTAATGGTGTTCGGTCCACTT |
| COX15 | CAGCGCCTAGAGCACAGTG | GCCAGACTCTGTCAACCTAGT |
| ATP5B | AAACAATTTGCTCCCATTCATGC | GACAACCTTGATACCAGTCACC |
| ATP5G1 | TTCCAGACCAGTGTTGTCTCC | GACGGGTTCCTGGCATAGC |
| ATP5G3 | ATGGTGTGTCTCAGCTAATCCA | CCACTCCTACTGTTGCAGCA |
| PAFAH1B3 | CGTGGCTGACAGCAAAGATA | GTCACCACCAATGCCAAAGT |
| SNW1 | GGATACCGGAAAGGCTGGATA | TGACTGTCCTTGTCGAGCAAT |
| SLC25A15 | CCTGAAGACTTACTCCCAGGT | GCGATGTTGGCGATTAGTGC |
| SLC25A22 | GCCAGCCAAGCTCATCAATG | GAGGCAGTCGGACATGCTC |
| NDUFA3 | GGGGCCTCGCTGTAATTCTG | GACGGGCACTGGGTAGTTG |
| UQCRC1 | GTTGGGATGGAGGTTGAATGA | GTGTGTATCTCTGTGCCTGTG |
| TIMM13 | CAGAGGATGACGGACAAGTGT | CATGTAGCGGTCCATGCACAIMMT |
| OPA1 | TGTGAGGTCTGCCAGTCTTTA | TGTCCTTAATTGGGGTCGTTG |
| TMPO | CCCCTCGGTCCTGACAAAAG | CGCTCTTCGTCACTGGAGAA |
| POLR2M | ACCATTGCGGACCAAGGTG | CGCATTTCTAGCACTTCCATGTA |
| β-ACTIN | CTCCATCCTGGCCTCGCTGT | GCTGTCACCTTCACCGTTCC |

Supplementary Table 3: Identified proteins that bind to HOMER3 in H1299 cells.

| Accession | Gene Name | Coverage [%] | # Peptides | # PSMs | # Unique Peptides | # AAs | MW [kDa] | calc. pI |
| --- | --- | --- | --- | --- | --- | --- | --- | --- |
| Q9NSC5 | HOMER3 | 81 | 33 | 314 | 31 | 361 | 39.8 | 5.45 |
| Q9Y6K5 | OAS3 | 1 | 1 | 1 | 1 | 1087 | 121.1 | 8.4 |
| Q16401 | PSMD5 | 2 | 1 | 1 | 1 | 504 | 56.2 | 5.48 |
| P62191 | PSMC1 | 3 | 1 | 1 | 1 | 440 | 49.2 | 6.21 |
| Q9Y3D9 | MRPS23 | 5 | 1 | 1 | 1 | 190 | 21.8 | 8.9 |
| Q96DV4 | MRPL38 | 3 | 1 | 1 | 1 | 380 | 44.6 | 7.53 |
| Q9BRJ2 | MRPL45 | 3 | 1 | 1 | 1 | 306 | 35.3 | 9.03 |
| P47914 | RPL29 | 9 | 1 | 1 | 1 | 159 | 17.7 | 11.66 |
| Q13085 | ACACA | 0 | 1 | 1 | 1 | 2346 | 265.4 | 6.37 |
| Q53FZ2 | ACSM3 | 2 | 1 | 1 | 1 | 586 | 66.1 | 9.04 |
| Q9BYF1 | ACE2 | 1 | 1 | 1 | 1 | 805 | 92.4 | 5.54 |
| Q01484 | ANK2 | 0 | 1 | 1 | 1 | 3957 | 433.4 | 5.14 |
| P09525 | ANXA4 | 2 | 1 | 1 | 1 | 319 | 35.9 | 6.13 |
| O43747 | AP1G1 | 1 | 1 | 1 | 1 | 822 | 91.3 | 6.8 |
| Q9BXS5 | AP1M1 | 2 | 1 | 1 | 1 | 423 | 48.6 | 7.3 |
| Q96CW1 | AP2M1 | 2 | 1 | 1 | 1 | 435 | 49.6 | 9.54 |
| O43776 | NARS1 | 2 | 1 | 1 | 1 | 548 | 62.9 | 6.25 |
| P61221 | ABCE1 | 2 | 1 | 1 | 1 | 599 | 67.3 | 8.34 |
| Q9UG63 | ABCF2 | 2 | 1 | 1 | 1 | 623 | 71.2 | 7.37 |
| Q9H2U1 | DHX36 | 1 | 1 | 1 | 1 | 1008 | 114.7 | 7.68 |
| Q92499 | DDX1 | 4 | 3 | 4 | 3 | 740 | 82.4 | 7.23 |
| Q9GZR7 | DDX24 | 1 | 1 | 1 | 1 | 859 | 96.3 | 9.06 |
| Q9BQ39 | DDX50 | 3 | 2 | 2 | 1 | 737 | 82.5 | 9.17 |
| Q8TDD1 | DDX54 | 1 | 1 | 1 | 1 | 881 | 98.5 | 10.02 |
| Q9UQB9 | AURKC | 3 | 1 | 1 | 1 | 309 | 35.6 | 8.81 |
| Q93088 | BHMT | 2 | 1 | 1 | 1 | 406 | 45 | 7.03 |
| Q9P287 | BCCIP | 3 | 1 | 1 | 1 | 314 | 36 | 4.61 |
| P51587 | BRCA2 | 0 | 1 | 1 | 1 | 3418 | 384 | 6.71 |
| O43670 | ZNF207 | 3 | 1 | 1 | 1 | 478 | 50.7 | 9.1 |
| P27708 | CAD | 2 | 4 | 4 | 4 | 2225 | 242.8 | 6.46 |
| Q13554 | CAMK2B | 2 | 1 | 1 | 1 | 666 | 72.6 | 7.27 |
| P50416 | CPT1A | 2 | 1 | 1 | 1 | 773 | 88.3 | 8.65 |
| P68400 | CSNK2A1 | 2 | 1 | 1 | 1 | 391 | 45.1 | 7.74 |
| Q8TDI0 | CHD5 | 1 | 1 | 1 | 1 | 1954 | 222.9 | 6.15 |
| P09496 | CLTA | 4 | 1 | 1 | 1 | 248 | 27.1 | 4.51 |
| Q8N684 | CPSF7 | 2 | 1 | 1 | 1 | 471 | 52 | 8 |
| P48444 | ARCN1 | 2 | 1 | 1 | 1 | 511 | 57.2 | 6.21 |
| O75534 | CSDE1 | 1 | 1 | 1 | 1 | 798 | 88.8 | 6.25 |
| Q9UBL6 | CPNE7 | 1 | 1 | 1 | 1 | 633 | 70.2 | 6.38 |
| P08574 | CYC1 | 5 | 1 | 1 | 1 | 325 | 35.4 | 9 |
| P47712 | PLA2G4A | 2 | 1 | 1 | 1 | 749 | 85.2 | 5.38 |
| Q5BKZ1 | ZNF326 | 2 | 1 | 1 | 1 | 582 | 65.6 | 5.15 |
| Q9Y295 | DRG1 | 4 | 1 | 1 | 1 | 367 | 40.5 | 8.9 |
| P52701 | MSH6 | 2 | 2 | 2 | 2 | 1360 | 152.7 | 6.9 |
| P33993 | MCM7 | 1 | 1 | 1 | 1 | 719 | 81.3 | 6.46 |
| P11387 | TOP1 | 1 | 1 | 1 | 1 | 765 | 90.7 | 9.31 |
| P11388 | TOP2A | 1 | 2 | 2 | 2 | 1531 | 174.3 | 8.72 |
| Q9UBS4 | DNAJB11 | 7 | 1 | 1 | 1 | 358 | 40.5 | 6.18 |
| P39656 | DDOST | 2 | 1 | 1 | 1 | 456 | 50.8 | 6.55 |
| Q8TCJ2 | STT3B | 3 | 3 | 3 | 3 | 826 | 93.6 | 8.91 |
| P55265 | ADAR | 1 | 1 | 1 | 1 | 1226 | 136 | 8.65 |
| Q13561 | DCTN2 | 5 | 1 | 1 | 1 | 401 | 44.2 | 5.21 |
| P50570 | DNM2 | 1 | 1 | 1 | 1 | 870 | 98 | 7.44 |
| P19474 | TRIM21 | 7 | 3 | 3 | 3 | 475 | 54.1 | 6.38 |
| Q9NZN4 | EHD2 | 2 | 1 | 1 | 1 | 543 | 61.1 | 6.46 |
| P38117 | ETFB | 3 | 1 | 1 | 1 | 255 | 27.8 | 8.1 |
| Q05639 | EEF1A2 | 25 | 10 | 12 | 1 | 463 | 50.4 | 9.03 |
| P24534 | EEF1B2 | 7 | 1 | 1 | 1 | 225 | 24.7 | 4.67 |
| P30040 | ERP29 | 4 | 1 | 1 | 1 | 261 | 29 | 7.31 |
| O94905 | ERLIN2 | 4 | 1 | 1 | 1 | 339 | 37.8 | 5.62 |
| Q9BY44 | EIF2A | 2 | 1 | 1 | 1 | 585 | 64.9 | 8.87 |
| Q9Y262 | EIF3L | 4 | 2 | 2 | 2 | 564 | 66.7 | 6.34 |
| P42285 | MTREX | 1 | 1 | 1 | 1 | 1042 | 117.7 | 6.52 |
| Q08945 | SSRP1 | 3 | 2 | 2 | 2 | 709 | 81 | 6.87 |
| Q96AE4 | FUBP1 | 9 | 5 | 5 | 5 | 644 | 67.5 | 7.61 |
| Q05397 | PTK2 | 1 | 1 | 1 | 1 | 1052 | 119.2 | 6.62 |
| Q96CW5 | TUBGCP3 | 1 | 1 | 1 | 1 | 907 | 103.5 | 8.12 |
| Q9UKL4 | GJD2 | 3 | 1 | 1 | 1 | 321 | 36.1 | 8.68 |
| Q9UKN8 | GTF3C4 | 1 | 1 | 1 | 1 | 822 | 91.9 | 6.65 |
| O94808 | GFPT2 | 1 | 1 | 1 | 1 | 682 | 76.9 | 7.37 |
| P09211 | GSTP1 | 5 | 1 | 1 | 1 | 210 | 23.3 | 5.64 |
| P11216 | PYGB | 9 | 5 | 5 | 5 | 843 | 96.6 | 6.86 |
| P49915 | GMPS | 2 | 1 | 1 | 1 | 693 | 76.7 | 6.87 |
| P04899 | GNAI2 | 4 | 1 | 1 | 1 | 355 | 40.4 | 5.54 |
| Q92598 | HSPH1 | 3 | 2 | 2 | 2 | 858 | 96.8 | 5.39 |
| P10412 | H1-4 | 31 | 9 | 13 | 2 | 219 | 21.9 | 11.03 |
| Q86YM7 | HOMER1 | 43 | 15 | 41 | 10 | 354 | 40.3 | 5.44 |
| Q9NSB8 | HOMER2 | 10 | 5 | 26 | 1 | 354 | 40.6 | 6.38 |
| Q16543 | CDC37 | 2 | 1 | 1 | 1 | 378 | 44.4 | 5.25 |
| Q96P70 | IPO9 | 1 | 1 | 1 | 1 | 1041 | 115.9 | 4.81 |
| Q14573 | ITPR3 | 0 | 1 | 1 | 1 | 2671 | 303.9 | 6.48 |
| P19525 | EIF2AK2 | 2 | 1 | 1 | 1 | 551 | 62.1 | 8.4 |
| Q13601 | KRR1 | 2 | 1 | 1 | 1 | 381 | 43.6 | 9.77 |
| P42166 | TMPO | 2 | 1 | 1 | 1 | 694 | 75.4 | 7.66 |
| O15230 | LAMA5 | 0 | 1 | 1 | 1 | 3695 | 399.5 | 7.02 |
| Q8WWI1 | LMO7 | 3 | 3 | 3 | 3 | 1683 | 192.6 | 8.09 |
| P31025 | LCN1 | 6 | 1 | 1 | 1 | 176 | 19.2 | 5.58 |
| Q9UNF1 | MAGED2 | 2 | 2 | 2 | 2 | 606 | 64.9 | 9.32 |
| Q13330 | MTA1 | 2 | 1 | 1 | 1 | 715 | 80.7 | 9.26 |
| Q8NCE2 | MTMR14 | 1 | 1 | 1 | 1 | 650 | 72.2 | 6.24 |
| Q9BYT8 | NLN | 1 | 1 | 1 | 1 | 704 | 80.6 | 6.64 |
| Q7Z417 | NUFIP2 | 2 | 1 | 1 | 1 | 695 | 76.1 | 8.7 |
| P80303 | NUCB2 | 2 | 1 | 1 | 1 | 420 | 50.2 | 5.12 |
| P78316 | NOP14 | 1 | 1 | 1 | 1 | 857 | 97.6 | 7.58 |
| P12270 | TPR | 0 | 1 | 1 | 1 | 2363 | 267.1 | 5.02 |
| Q5JVF3 | PCID2 | 6 | 1 | 3 | 1 | 399 | 46 | 8.53 |
| Q13162 | PRDX4 | 11 | 4 | 4 | 1 | 271 | 30.5 | 6.29 |
| Q9BQI7 | PSD2 | 1 | 1 | 1 | 1 | 771 | 84.6 | 5.19 |
| Q9NSD9 | FARSB | 5 | 3 | 3 | 3 | 589 | 66.1 | 6.84 |
| Q15102 | PAFAH1B3 | 4 | 1 | 1 | 1 | 231 | 25.7 | 6.84 |
| Q9UHX1 | PUF60 | 3 | 1 | 1 | 1 | 559 | 59.8 | 5.29 |
| Q86U42 | PABPN1 | 9 | 2 | 2 | 2 | 306 | 32.7 | 5.06 |
| O95758 | PTBP3 | 2 | 1 | 1 | 1 | 552 | 59.7 | 9.04 |
| O94906 | PRPF6 | 2 | 2 | 2 | 2 | 941 | 106.9 | 8.25 |
| Q6P2Q9 | PRPF8 | 3 | 7 | 7 | 7 | 2335 | 273.4 | 8.84 |
| Q92620 | DHX38 | 1 | 1 | 1 | 1 | 1227 | 140.4 | 6.54 |
| Q8IY81 | FTSJ3 | 1 | 1 | 1 | 1 | 847 | 96.5 | 8.4 |
| Q9BUQ8 | DDX23 | 1 | 1 | 1 | 1 | 820 | 95.5 | 9.55 |
| Q96GQ7 | DDX27 | 3 | 2 | 2 | 2 | 796 | 89.8 | 9.28 |
| Q9H0S4 | DDX47 | 2 | 1 | 1 | 1 | 455 | 50.6 | 9.1 |
| P28370 | SMARCA1 | 1 | 2 | 2 | 2 | 1054 | 122.5 | 8.09 |
| Q4AC99 | ACCSL | 1 | 1 | 1 | 1 | 568 | 65.2 | 6.49 |
| P12004 | PCNA | 15 | 3 | 3 | 3 | 261 | 28.8 | 4.69 |
| P49720 | PSMB3 | 8 | 1 | 1 | 1 | 205 | 22.9 | 6.55 |
| Q9UKV8 | AGO2 | 1 | 1 | 1 | 1 | 859 | 97.1 | 9.19 |
| Q96G01 | BICD1 | 1 | 1 | 1 | 1 | 975 | 110.7 | 5.81 |
| Q9UKK3 | PARP4 | 7 | 9 | 9 | 9 | 1724 | 192.5 | 5.66 |
| P35813 | PPM1A | 10 | 4 | 6 | 1 | 382 | 42.4 | 5.36 |
| Q14690 | PDCD11 | 1 | 2 | 2 | 2 | 1871 | 208.6 | 8.87 |
| P18583 | SON | 0 | 1 | 1 | 1 | 2426 | 263.7 | 5.64 |
| Q9NYQ8 | FAT2 | 0 | 1 | 1 | 1 | 4349 | 479 | 5.16 |
| Q9P2R3 | ANKFY1 | 1 | 1 | 1 | 1 | 1169 | 128.3 | 6.1 |
| P59190 | RAB15 | 5 | 1 | 1 | 1 | 212 | 24.4 | 5.71 |
| Q8TDY2 | RB1CC1 | 1 | 1 | 1 | 1 | 1594 | 183 | 5.41 |
| Q92900 | UPF1 | 1 | 1 | 1 | 1 | 1129 | 124.3 | 6.61 |
| Q5T5U3 | ARHGAP21 | 1 | 2 | 2 | 2 | 1958 | 217.3 | 7.8 |
| Q99575 | POP1 | 1 | 1 | 1 | 1 | 1024 | 114.6 | 9.22 |
| Q8TDN6 | BRIX1 | 2 | 1 | 1 | 1 | 353 | 41.4 | 9.92 |
| Q08J23 | NSUN2 | 1 | 1 | 1 | 1 | 767 | 86.4 | 6.77 |
| Q96PK6 | RBM14 | 4 | 2 | 2 | 2 | 669 | 69.4 | 9.67 |
| Q9NW13 | RBM28 | 1 | 1 | 1 | 1 | 759 | 85.7 | 9.22 |
| P52756 | RBM5 | 5 | 4 | 4 | 2 | 815 | 92.1 | 6.28 |
| Q8WXA3 | RUFY2 | 1 | 1 | 1 | 1 | 606 | 70 | 5.83 |
| Q14BN4 | SLMAP | 1 | 1 | 1 | 1 | 828 | 95.1 | 5.47 |
| Q8WVM8 | SCFD1 | 2 | 1 | 1 | 1 | 642 | 72.3 | 6.27 |
| P04279 | SEMG1 | 2 | 1 | 2 | 1 | 462 | 52.1 | 9.29 |
| Q92599 | SEPTIN8 | 2 | 1 | 1 | 1 | 483 | 55.7 | 6.28 |
| Q13242 | SRSF9 | 16 | 3 | 3 | 3 | 221 | 25.5 | 8.65 |
| Q9Y6E0 | STK24 | 2 | 1 | 1 | 1 | 443 | 49.3 | 5.69 |
| Q9BXP5 | SRRT | 1 | 1 | 1 | 1 | 876 | 100.6 | 5.96 |
| Q9UHB9 | SRP68 | 2 | 1 | 1 | 1 | 627 | 70.7 | 8.56 |
| Q13573 | SNW1 | 3 | 1 | 1 | 1 | 536 | 61.5 | 9.52 |
| P02549 | SPTA1 | 1 | 1 | 1 | 1 | 2419 | 279.8 | 5.05 |
| Q14247 | CTTN | 4 | 2 | 2 | 2 | 550 | 61.5 | 5.4 |
| Q96SB4 | SRPK1 | 2 | 1 | 1 | 1 | 655 | 74.3 | 6.16 |
| P78362 | SRPK2 | 2 | 1 | 1 | 1 | 688 | 77.5 | 4.97 |
| P31948 | STIP1 | 7 | 4 | 4 | 4 | 543 | 62.6 | 6.8 |
| A6NHR9 | SMCHD1 | 1 | 1 | 1 | 1 | 2005 | 226.2 | 7.3 |
| Q14683 | SMC1A | 1 | 1 | 1 | 1 | 1233 | 143.1 | 7.64 |
| Q9NPQ8 | RIC8A | 2 | 1 | 1 | 1 | 531 | 59.7 | 5.33 |
| Q92804 | TAF15 | 6 | 1 | 1 | 1 | 592 | 61.8 | 8.02 |
| Q68CZ2 | TNS3 | 1 | 1 | 1 | 1 | 1445 | 155.2 | 6.81 |
| Q96HA7 | TONSL | 1 | 1 | 1 | 1 | 1378 | 150.8 | 6.42 |
| Q9Y4P3 | TBL2 | 2 | 1 | 1 | 1 | 447 | 49.8 | 9.44 |
| P22102 | GART | 2 | 2 | 2 | 2 | 1010 | 107.7 | 6.7 |
| P06753 | TPM3 | 14 | 4 | 5 | 1 | 285 | 32.9 | 4.72 |
| Q9UIG0 | BAZ1B | 1 | 1 | 1 | 1 | 1483 | 170.8 | 8.48 |
| P18031 | PTPN1 | 3 | 1 | 1 | 1 | 435 | 49.9 | 6.27 |
| O43290 | SART1 | 3 | 2 | 2 | 2 | 800 | 90.2 | 6.13 |
| Q93009 | USP7 | 1 | 1 | 1 | 1 | 1102 | 128.2 | 5.55 |
| A6NNT2 | C16orf96 | 1 | 2 | 2 | 1 | 1141 | 125 | 7.03 |
| Q9UM54 | MYO6 | 1 | 1 | 1 | 1 | 1294 | 149.6 | 8.53 |
| Q16851 | UGP2 | 2 | 1 | 1 | 1 | 508 | 56.9 | 8.15 |
| O14975 | SLC27A2 | 2 | 1 | 1 | 1 | 620 | 70.3 | 8.51 |
| Q00341 | HDLBP | 1 | 1 | 1 | 1 | 1268 | 141.4 | 6.87 |
| Q7Z5H4 | VN1R5 | 2 | 1 | 1 | 1 | 357 | 40.8 | 9.2 |
| P67809 | YBX1 | 16 | 4 | 7 | 1 | 324 | 35.9 | 9.88 |
| P49750 | YLPM1 | 1 | 1 | 1 | 1 | 2146 | 241.5 | 6.55 |
| Q8N680 | ZBTB2 | 3 | 1 | 1 | 1 | 514 | 57.3 | 6.1 |
| P17031 | ZNF26 | 2 | 1 | 1 | 1 | 533 | 61.2 | 8.84 |
